# *In vivo* molecular imaging of breast cancer metabolic heterogeneity using [1-^13^C]pyruvate-d_3_ hyperpolarized by reversible exchange with parahydrogen

**DOI:** 10.7150/thno.103272

**Published:** 2025-03-03

**Authors:** Stefan Petersen, Luca Nagel, Philipp R. Groß, Henri de Maissin, Robert Willing, Lisa Heß, Julia Mitschke, Nicole Klemm, Judith Treiber, Christoph A. Müller, Stephan Knecht, Ilai Schwartz, Moritz Weigt, Michael Bock, Dominik von Elverfeldt, Maxim Zaitsev, Eduard Y. Chekmenev, Jan-Bernd Hövener, André F. Martins, Franz Schilling, Thomas Reinheckel, Andreas B. Schmidt

**Affiliations:** 1 Division of Medical Physics, Department of Radiology, University Medical Center Freiburg, Faculty of Medicine, University of Freiburg, Killianstr. 5a, Freiburg 79106, Germany.; 2 German Cancer Consortium (DKTK), partner site Freiburg, a partnership between DKFZ and University Medi-cal Center Freiburg, Germany.; 3 Department of Nuclear Medicine, TUM School of Medicine and Health, TUM University Hospital, Technical University of Munich, Munich, Germany, Ismaninger Str. 22, Munich 81675, Germany.; 4 Institute of Molecular Medicine and Cell Research, Faculty of Medicine, University of Freiburg, Stefan-Meier-Str. 17, Freiburg 79104, Germany.; 5 NVision Imaging Technologies GmbH, Wolfgang-Paul-Str. 2, Ulm 89081, Germany.; 6 Department of Chemistry, Integrative Biosciences (Ibio), Karmanos Cancer Institute (KCI), Wayne State University, Detroit, Michigan 48202, United States; 7 Section Biomedical Imaging, Molecular Imaging North Competence Center MOINCC, Department of Radiology and Neuroradiology, University Hospital Schleswig Holstein, Kiel University, Germany.; 8 Werner Siemens Imaging Center, Department of Preclinical Imaging and Radiopharmacy, Eberhard Karls University Tübingen, Röntgenweg 13, Tübingen 72076, Germany.; 9 Cluster of Excellence iFIT (EXC 2180) "Image-Guided and Functionally Instructed Tumor Therapies", Eberhard Karls University Tübingen, Germany.; 10 German Cancer Consortium (DKTK), partner site Tubingen, a partnership between DKFZ and University Hos-pital Tubingen, Germany.; 11 Centre for Biological Signalling Studies BIOSS, University of Freiburg, Freiburg 79104, Germany.; 12 Munich Institute of Biomedical Engineering, Technical University of Munich, Garching, Germany, Boltzmannstr. 11, Garching 85748, Germany.; 13 German Cancer Consortium (DKTK), partner site Munich, a partnership between DKFZ and University Hospi-tal Munich, Germany.

**Keywords:** parahydrogen, hyperpolarization, SABRE, metabolic imaging, MMTV-PyMT

## Abstract

Metabolic MRI using hyperpolarized (HP) [1-^13^C]pyruvate is promising for diagnostic medicine, allowing the study of cancer metabolism and early detection of therapy response. However, a possible widespread and routine use requires high-throughput and user-friendly technologies to produce the hyperpolarized media.

**Methods:** Recently, we introduced a fast (6 min) and cost-effective method using Spin-Lock Induced Crossing and Signal Amplification By Reversible Exchange (SLIC-SABRE) at µT fields and a rapid purification to produce biocompatible HP solutions of aqueous pyruvate. In this study, we used SLIC-SABRE to conduct *in vivo* tumor metabolic imaging in a transgenic breast cancer mouse model (MMTV-PyMT).

**Results:** In agreement with previous HP MRI cancer studies, an elevated lactate metabolism was found in tumors compared to healthy breast tissue, heart, and vasculature, as well as distinct metabolic profiles within different tumor compartments. These findings suggest a potential link between lactate-to-pyruvate ratios and the varying levels of tumor cell proliferation and apoptosis observed by histological analyses.

**Conclusion:** Our results underscore the potential of SABRE to enhance the accessibility and throughput of HP MRI, thereby advancing cancer research and diagnostic oncology.

## Introduction

Cancer remains a significant challenge in modern medicine, with breast cancer representing one of the most prevalent and heterogeneous malignancies 1. Despite incremental advancements in therapies, including surgery, radiation, and targeted therapies, there remains an urgent medical need to improve clinical outcomes. The complexity of cancer, underscored by its molecular diversity and intratumoral heterogeneity, necessitates personalized approaches for accurate diagnosis and effective treatment guided by non-invasive *in vivo* imaging 2.

Imaging technologies are playing a pivotal role in cancer care by providing non-invasive assessments of tumor characteristics and treatment responses. Traditional anatomical imaging techniques often cannot provide molecular-level information on cancer development. To address this limitation, molecular imaging techniques have emerged with breakthrough applications 3,4.

Hyperpolarized magnetic resonance imaging (HP MRI) allows for transient signal enhancements of over 10,000-fold 5. HP MRI offers non-invasive insights into biochemical pathways *in vivo* without ionizing radiation. HP MRI is the only technology that enables the visualization of metabolic conversion *in vivo* with spatial and real-time temporal resolution. HP [1-^13^C]-pyruvate showed great promise for investigating cancer as it is a central metabolite in energy metabolic pathways at the intersection of glycolysis and the TCA cycle. Pyruvate has been used to image metabolism in many cancers, including prostate cancer 6, glioblastoma 7, and bone and liver metastases 8, providing unprecedented insights into tumor biology. Clinically, detecting the Warburg effect - enhanced glycolysis and lactate formation even in the presence of high oxygen levels in tumors - provides essential information regarding tumor grading 9 and active surveillance of therapy response in oncological patients 10-12. Lactate, the lactate-to-pyruvate ratio and the *k*_PL_ value were identified as promising biomarkers for tumor aggressiveness, grading and therapy response 9,13-15. HP [1-^13^C]-pyruvate MRI is under investigation in over 50 clinical trials 4.

The implementation of HP ^13^C MRI is currently impeded by several challenges, hindering its widespread application and clinical translation. Notably, these include the short lifetime of the hyperpolarized agent, high cost, the large size of the equipment, low throughput, and the requirement for highly trained personnel, all of which contribute to its slow development.

Dissolution Dynamic Nuclear Polarization (dDNP) is the leading technology for producing HP [1-^13^C]-pyruvate 16. It requires strong magnetic fields (typically 5-7 T), cryogenic temperatures (<2 K), and microwave irradiation to polarize agents in the solid phase, followed by rapid thawing and dissolution with superheated water to transfer HP metabolites into the liquid phase. Preparing HP boluses is elaborate and time-consuming, taking about an hour for a bolus of HP [1-^13^C]pyruvate; however, many molecular probes can be polarized with dDNP 12,17. Impressive results of HP MRI have driven ongoing efforts to improve dDNP 18-21 and develop alternative technologies for faster, more versatile, and cost-effective HP media production 22,23.

Parahydrogen-induced polarization (PHIP) 24,25 technologies offer a solution to several challenges by providing rapid hyperpolarization with simple, low-footprint hardware and reduced production costs 22. PHIP involves the permanent addition of parahydrogen to the substrate through direct or side-arm hydrogenation (PHIP-SAH) 26. This method, particularly with HP [1-^13^C]pyruvate, has been used in *in vivo* cardiac 27 and tumor studies 28-30 in mice and is being developed for commercial preclinical and clinical devices. However, identifying and synthesizing suitable unsaturated precursors is often challenging and costly. Additionally, the chemical modification (i.e., hydrogenation) done seconds before the *in vivo* administration introduces regulatory complexities and may require manufacturing licenses for clinical applicants in some countries 22.

A non-hydrogenative PHIP referred to as SABRE (Signal Amplification By Reversible Exchange) has been presented by Duckett and coworkers 23,31,32. SABRE employs a reversible exchange of parahydrogen and suitable substrates with an iridium catalyst 33, allowing for continuous polarization transfer without altering the chemical structure of the substrate 34,35. Therefore, the method combines the advantages of PHIP - such as speed, low cost, and simplicity - with some of the flexibility of dDNP, which has enabled the ^13^C and ^15^N SABRE hyperpolarization of multiple agents 23,36-38, including [1-^13^C]-pyruvate 32,39-42. Consequently, the translation of SABRE to preclinical studies and clinical settings is highly promising. Recently, SABRE-SHEATH with a fluorinated catalyst and [1-^13^C]pyruvate-h_3_ was used to perform the first ^13^C-MR spectroscopy of mice with pancreatic cancer xenografts 43.

**Figure 1:**
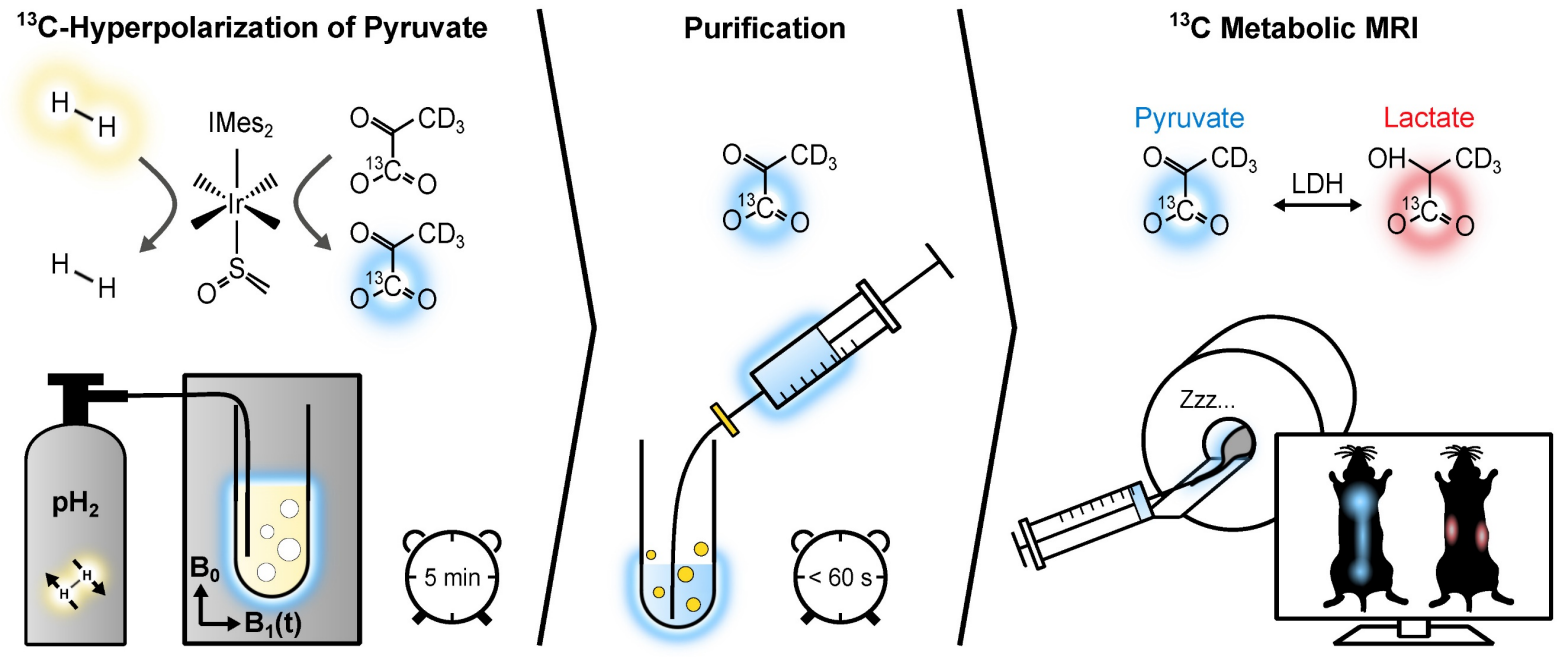
The experimental protocol for *in vivo* metabolic MRI. Initially, [1-^13^C]-pyruvate-*d*_3_ undergoes hyperpolarization via reversible exchange with parahydrogen at an iridium complex. Parahydrogen is bubbled through an NMR tube containing the SABRE reaction solution while applying a 2 µT SLIC radiofrequency field in a 50 µT static magnetic field (left). Subsequently, the hyperpolarized [1-^13^C]-pyruvate-*d*_3_ is transferred from methanol-*d*_4_ to a PBS of D_2_O, and the resulting solution is collected in a syringe after filtration to remove the non-water-soluble iridium complex (center). This biocompatible [1-^13^C]-pyruvate-*d*_3_ solution is then administered to the tumor-bearing mice, allowing for the monitoring of its conversion into downstream metabolic products, i.e., [1-^13^C]-lactate-*d*_3_ (right).

We have introduced a fast and cost-effective method using SABRE and Spin-Lock Induced Crossing (SLIC-SABRE) 44,45 at microtesla fields 46,47, which can polarize [1-^13^C]pyruvate-*d*_3_ to over 20% in methanol-d_4_, sufficient for ^13^C MRI. Initially, this was achieved in a toxic methanol solution containing the iridium-based exchange catalyst. However, a rapid purification process has enabled us to produce HP solutions of aqueous pyruvate suitable for *in vivo* applications, **Figure 1** 48. The deuteration of pyruvate results in an increased polarization lifetime without affecting the kinetics of pyruvate metabolism 46,49.

In the present study, we demonstrate ^13^C MRI of cancer using aqueous [1-^13^C]pyruvate-*d*_3_ hyperpolarized using SLIC-SABRE. Focusing on transgenic breast cancer mice, we reveal considerable lactate production and pronounced intra- and intertumoral heterogeneity of the lactate metabolism, which correlates well with histological results.

## Methods

50 mM [1-^13^C]-pyruvate-*d*_3_ was hyperpolarized to up to 24% in 600 µL methanol-*d*_4_ using SLIC-SABRE at a 50 µT static magnetic field. Subsequently, 600 µL phosphate-buffered solution (PBS) of D_2_O was added and the sample was evacuated to evaporate methanol-*d*_4_ at approximately 100 mT, 5 mbar, and 100 °C (250 µM residual methanol). The aqueous solution was then filtered to remove the precipitated iridium catalyst, resulting in [1-^13^C]-pyruvate-*d*_3_ in D_2_O solution of pH 6.5 - 7, ready for administration to the mice, **Figure 1**. This way, we efficiently produced a batch of 30 mM aqueous, pH-neutral HP [1-^13^C]-pyruvate-*d*_3_, hyperpolarized up to *P*_13C_ ≈ 10% in less than 6 minutes, **Figure S1**.

To investigate the efficacy of SABRE hyperpolarized pyruvate in assessing tumor-specific metabolic processes *in vivo*, a transgenic breast cancer model (mouse mammary tumor virus - polyomavirus middle tumor-antigen; MMTV-PyMT) was employed 15,50,51. This viral oncogene activates various oncogenic signals in the mammary epithelium. Most notably, it hyper-activates the phosphoinositide 3-kinase (PI3K) pathway, mimicking a common characteristic observed in approximately 70% of breast cancer patients. The onset of mammary adenomas in MMTV-PyMT mice typically occurs at about 6 weeks of age, rapidly progressing to large, invasive, and poorly differentiated carcinomas within 8 weeks. The numbering of breast tumors and mammary glands is shown in **Figure S2**.

Ethical approval for the animal experiments was obtained from the relevant authority (Regierungspraesidium Freiburg, Talstr. 4-8, 79095 Freiburg; AZ: 35-9185.81/G-21/134). Vital parameters of the rodents were continuously monitored throughout the MRI procedure (see Supporting Information (SI) for details). A cohort of six MMTV-PyMT mice (age: 10-14 weeks, weight: approximately 24 g) exhibiting various tumor stages were investigated.

For MRI, a dedicated mouse ^1^H/^13^C quadrature resonator (inner diameter of 3.5 cm, V-XQ-HQ-070-02222, Rapid Biomedical GmbH, Germany) and a 7 T preclinical MRI system (Biospec 70/20, PV6.0.1, Bruker, Germany) were utilized. Following the injection of a single bolus of a 30mM HP [1-^13^C]-pyruvate-*d*_3_ solution through the tail vein (≤5 µL/g mouse weight), ^13^C images were acquired using center-out phase encoded free-induction decay chemical shift imaging (FID-CSI) to monitor pyruvate-to-lactate conversion. The FID-CSI sequence was chosen for its known robustness, despite limitations like a broad point spread function (PSF) at small matrix sizes and inefficient use of the hyperpolarized state due to per-phase encode excitation. This study is the first to use SLIC SABRE for detecting tumor heterogeneity, valuing localized spectroscopic imaging to assess local metabolism without introducing uncertainties from sequence design or complex data analysis. Region-of-interest-based metabolite time curves showed varied profiles, indicating the broad PSF has minimal impact. For data processing, pyruvate and lactate images were generated by time-domain fitting of Free Induction Decay (FID) signals for multiple metabolites (pyruvate, lactate, alanine, pyruvate hydrate) using Python 3.10, minimizing the sum-of-squares difference between measured and modeled data (see supplementary **Figure S3**). For dynamic CSI, images were generated by averaging fitted FID amplitudes across repetitions and then showing the absolute values of the metabolite amplitudes, while single-repetition acquisitions used the absolute amplitudes directly. ROI spectra were computed as weighted averages of magnitude spectra based on voxel coverage within the ROI (see supplementary **Figure S4**). Averaging was done by averaging the absolute spectra across the ROI to avoid signal cancellation due to phase differences. Further experimental details and non-interpolated ^13^C images (see supplementary **Figure S13**) are provided in the SI.

## Results and Discussion

Metabolic imaging revealed prominent lactate metabolism within tumor regions, showcasing the heterogeneous nature of the cancers both anatomically and metabolically. Metabolic imaging results from three of these mice are presented in subsequent sections, while the remaining investigations, along with metabolite signal evolution from all experiments, are provided in the SI.

### Mouse A - Localized Metabolic Imaging

**Figure 2:**
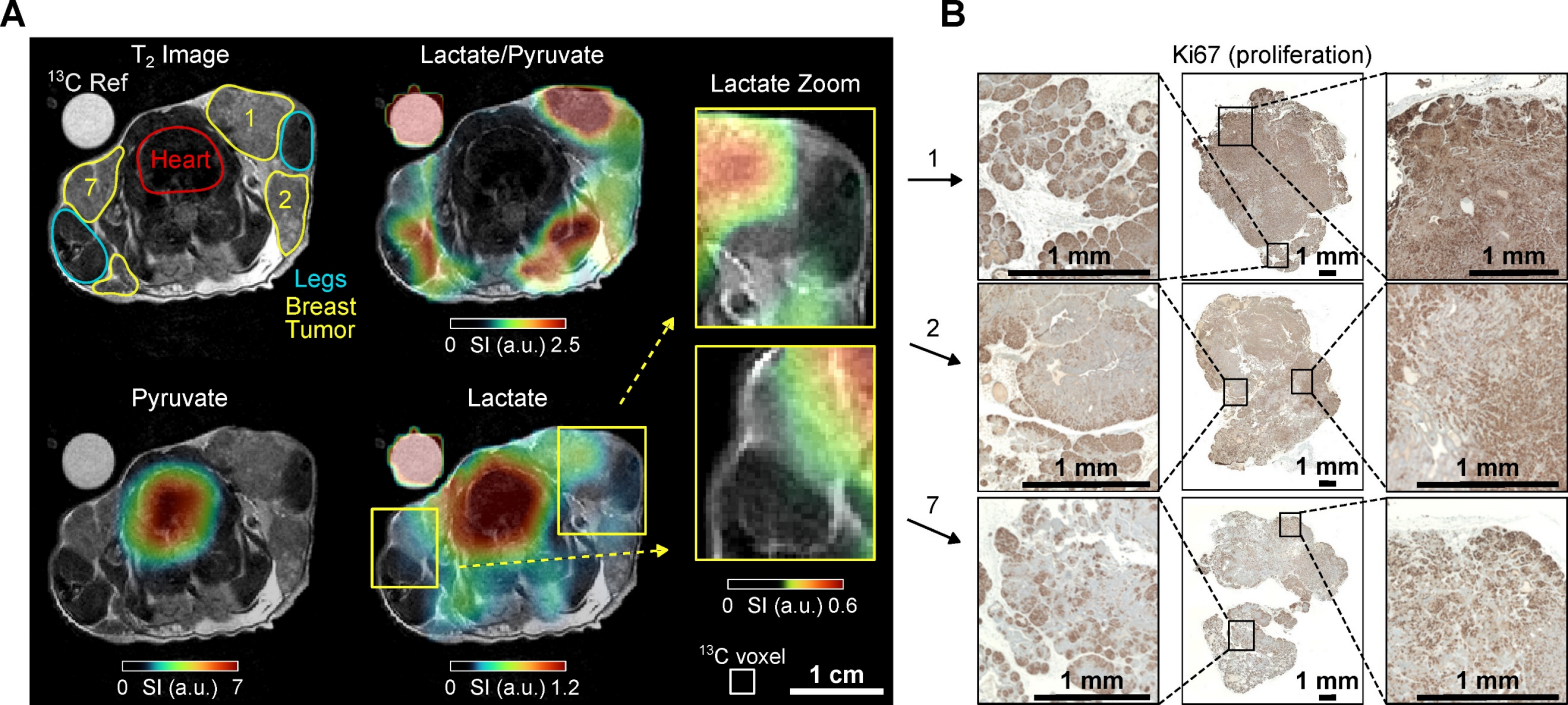
*In vivo* ^1^H T_2_w MRI and dynamic ^13^C chemical shift imaging of PyMT mouse A after administering SABRE-hyperpolarized [1-^13^C]-pyruvate-d_3,_ and histology (Ki67 staining) of the imaged tumors. (**A**) Axial anatomic ^1^H MRI showing the tumors on mammary glands 1, 2 and 7, heart, legs, and a ^13^C-lactate reference (left). The anatomic slice is superimposed with time-summated and interpolated ^13^C-lactate/^13^C-pyruvate (top right), ^13^C-pyruvate (bottom left) and ^13^C-lactate signal (bottom right). The lactate signal is enhanced in the breast tumors compared to other non-cancerous tissue (e.g., leg muscle). Note that low lactate signals can lead to high lactate-to-pyruvate ratios without significance if the pyruvate signal in the same voxel is negligible (no thresholding was applied to the ratio maps). The apparently elevated 13C signals in the leg and decreased lactate signal in gland 2 can be attributed to partial volume effects and slice positioning, as detailed in Figure S5. The regions indicated as leg and tumor do not exclusively correspond to these anatomical structures within the entire ^13^C imaging slice. The white box (bottom left) indicates the native CSI-FID resolution. (**B**) Histology of all three breast tumors with Ki67 staining indicates cell proliferation (brown). Remarkably, the tumor on mammary gland 1 has a significantly higher lactate-to-pyruvate ratio and appears to be more proliferative than other tumors.

Mouse A (age: 13 weeks, weight 24 g) with localized nodules characterized by palpable tumors in all 10 mammary glands. For MRI analysis, an axial region of interest (ROI) was selected that included mammary glands 1, 2, and 7, the lungs, the lower heart ventricles, and the forelimbs, **Figure 2**.

Anatomical ^1^H MRI revealed distinct tumorous nodes with varying sizes surrounding the forelimbs. The largest observed diameter of a single mammary carcinoma was ≈ 13 mm at gland 1. Most tumor areas exhibited similar T_2_w intensity despite differences in node size, **Figure 2**.

Intense lactate signals were observed in the tumors. Although the native ^13^C MRI resolution is limited, the muscles, particularly the front legs, were still distinguishable from the surrounding tumor tissue in the ^13^C-lactate images, **Figure 2**. Large pyruvate and lactate signals were also observed in the heart, but the lactate-to-pyruvate ratio is negligible compared to the other regions. Analysis of the signal-time curves detected the arrival of the pyruvate bolus and subsequent formation of downstream lactate in the tumor and, to a lesser extent, in the heart, **Figure S5**. Interestingly, when comparing the tumors, the tumor on gland 1 exhibits higher lactate levels and lactate-to-pyruvate ratios compared to the tumors on glands 2 and 7. This observation aligns with the corresponding histology, where Ki67 staining indicates higher cell proliferation levels in tumor 1. In contrast, tumor 7 shows slightly elevated apoptosis and reduced proliferation (see supplementary **Figures S5** and** S15**), which may explain the reduced pyruvate-to-lactate conversion, as reflected by the lower kPL​ value and lactate-to-pyruvate ratio (see supplementary **Figure S12** and **Table S1**). Note that the high lactate-to-pyruvate (Lac/Pyr) ratio observed in the legs can be attributed, at least in part, to the low pyruvate signal, which is close to the noise level, making the calculated ratio less reliable in these regions. Additionally, due to the 5-mm slice thickness used for the ^13^C MRI, tumors 1 and 7 partially extend into the area labeled as 'leg' in the displayed 1H MRI. This overlap is not apparent in the 1H MRI, which was acquired with thinner slices (see supplementary **Figure S5G**). The metabolic contributions of these tumors, combined with partial volume effects, likely account for the elevated ^13^C Lac/Pyr ratio observed in the leg regions near the tumors.

### Mouse B - Imaging tumor heterogeneity

**Figure 3:**
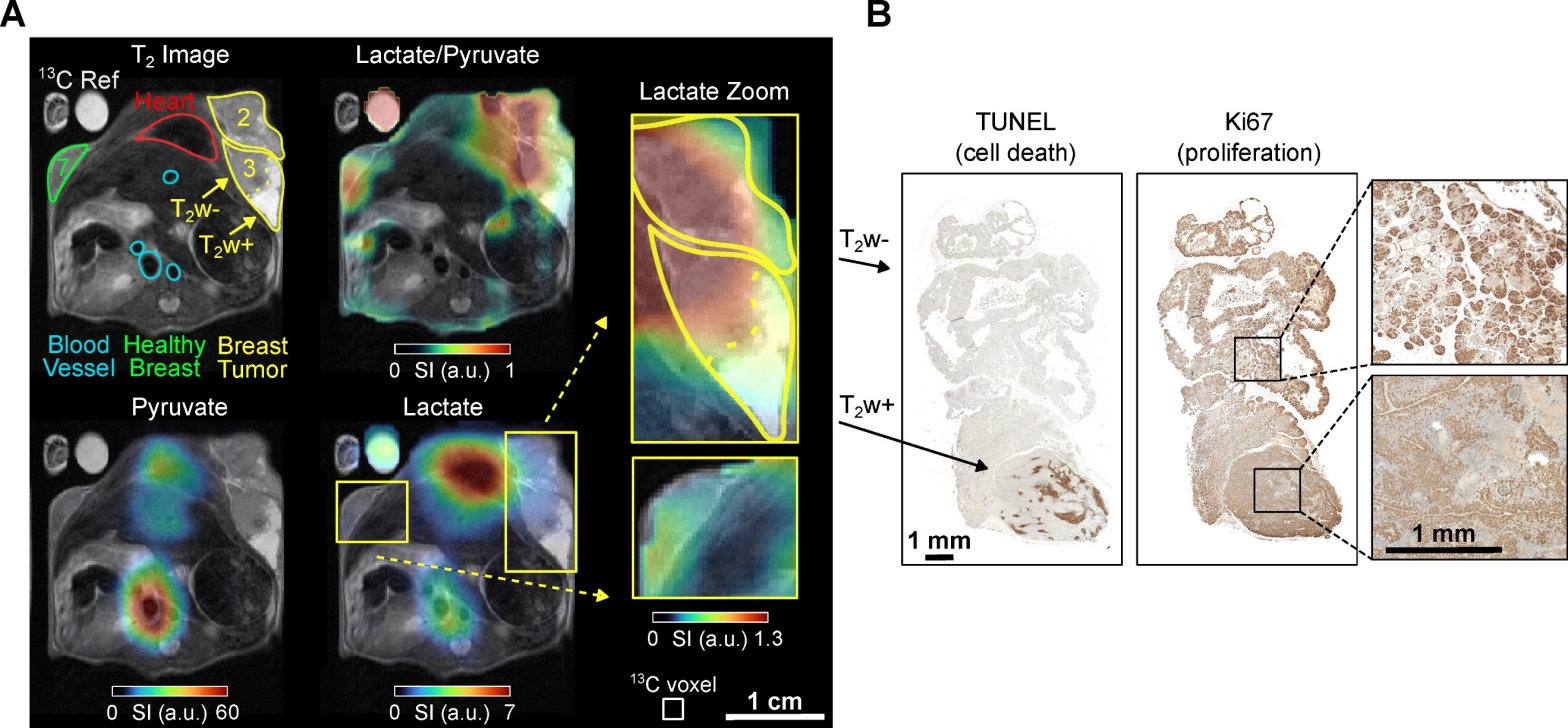
*In vivo* ^1^H T_2_w MRI and ^13^C chemical shift imaging of PyMT mouse B after administering SABRE-hyperpolarized [1-^13^C]-pyruvate-d_3_ and histology (Ki67 staining) of the imaged tumors. (**A**) Axial anatomic ^1^H MRI showing the tumors on mammary glands 2 and 3, non-cancerous appearing breast tissue on gland 7, heart, blood vessels, and a ^13^C-lactate reference (left). The anatomic slice is superimposed with the interpolated ^13^C-lactate/^13^C-pyruvate (top right), ^13^C-pyruvate (bottom left) and ^13^C-lactate signal (bottom right). The lactate signal is enhanced in the breast tumors (2 and 3) compared to the non-cancerous appearing breast tissue on mammary gland 7. The heterogeneous breast tumor on mammary gland 3 divides into a hyper-intense (T_2_w+) region with lower lactate and lactate-to-pyruvate signals and a hypo-intense (T_2_w-) tumor region with higher signals. The pyruvate signal was received from all voxels in the reported regions of interest, see Fig. S6, but is less visible here because of the color scale windowing applied in presence of the strong metabolite signals in the heart and main vessels. Note that histology suggested that the tumors were well vascularized and perfused (see SI Fig. S14). The white box indicates the native CSI-FID resolution. (**B**) Histology of the breast tumor with TUNEL staining indicating ongoing apoptosis in the T_2_w+ region (left) and Ki67 staining indicating cell proliferation in the T_2_w- region (right).

Mouse B (age: 12 weeks, weight 24 g) presented with advanced breast cancer characterized by palpable smaller tumors in mammary glands 4 and 9, and larger nodes in glands 2 and 3. No tumor was palpable in the contralateral mammary gland 7. Hence, a cross-section of the upper chest covering mammary glands 2, 3, and 7 was subjected to MRI analysis to examine differences in tumorous and healthy-appearing breast tissue, **Figure 3**.

Anatomical ^1^H MRI revealed the tumorous nodes 2 and 3, with approximate maximum diameters of ≈10-12 mm, respectively. Interestingly, while 2 and 3 were similar in size, 3 exhibited a conspicuous tumor area with enhanced intensity in the T_2_-weighted MRI images (hyper-intense, T_2_w+). Anatomical MRI revealed no apparent abnormalities in gland 7, which additionally appeared significantly smaller compared to 2 and 3, **Figure 3**.

The HP ^13^C MRI data showed the strongest ^13^C pyruvate and lactate signals in the heart. The lactate level was elevated in the tumor compared to the contralateral non-cancerous appearing breast tissue (gland 7). The lactate-to-pyruvate ratio map provides additional evidence that the tumor is the primary source of the lactate signal, as the high ratio is most pronounced within the tumor region and at the interface between the tumor and the heart. The T_2_w+ region exhibited lower pyruvate and lactate signals than the hypo-intense (T_2_w-) region, suggesting lower metabolic activity and perfusion, **Figure 3**. It should be noted that this data was recorded statically at a single time point that started 10 seconds post-injection. At this time, metabolic conversion of pyruvate to lactate was not much advanced, resulting in overall low lactate signals throughout the body compared to the other experiments reported.

The possible correlation between T_2_w+ and T_2_w- regions and histology is particularly evident in this mouse, **Figure 3B**. The T_2_w- area of tumor B exhibits a high proportion (~13%) of necrotic or apoptotic cells, as determined by TUNEL staining, compared to the T_2_w+ region, which shows less than 1% cell death (see supplementary **Figure S15**). In contrast, markers of proliferation (Ki67 staining) and vascularization (CD31 staining, see supplementary **Figure S14**) appear unchanged between T_2_w hypo- and hyperintense regions. Notably, the Lac/Pyr ratio and overall ^13^C signal were significantly reduced in the T_2_w- area, suggesting a potential association with a higher proportion of non-viable cells.

### Mouse C - Imaging tumor heterogeneity

Mouse C (age: 13 weeks, weight 26.7 g) also presented with tumors at mammary glands 1-3, and smaller tumors at 6 and 7. Again, we examined an ROI in the upper body, but the imaging slice was higher positioned compared to the previous experiments, excluding the heart but including a portion of the mouse brain.

**Figure 4:**
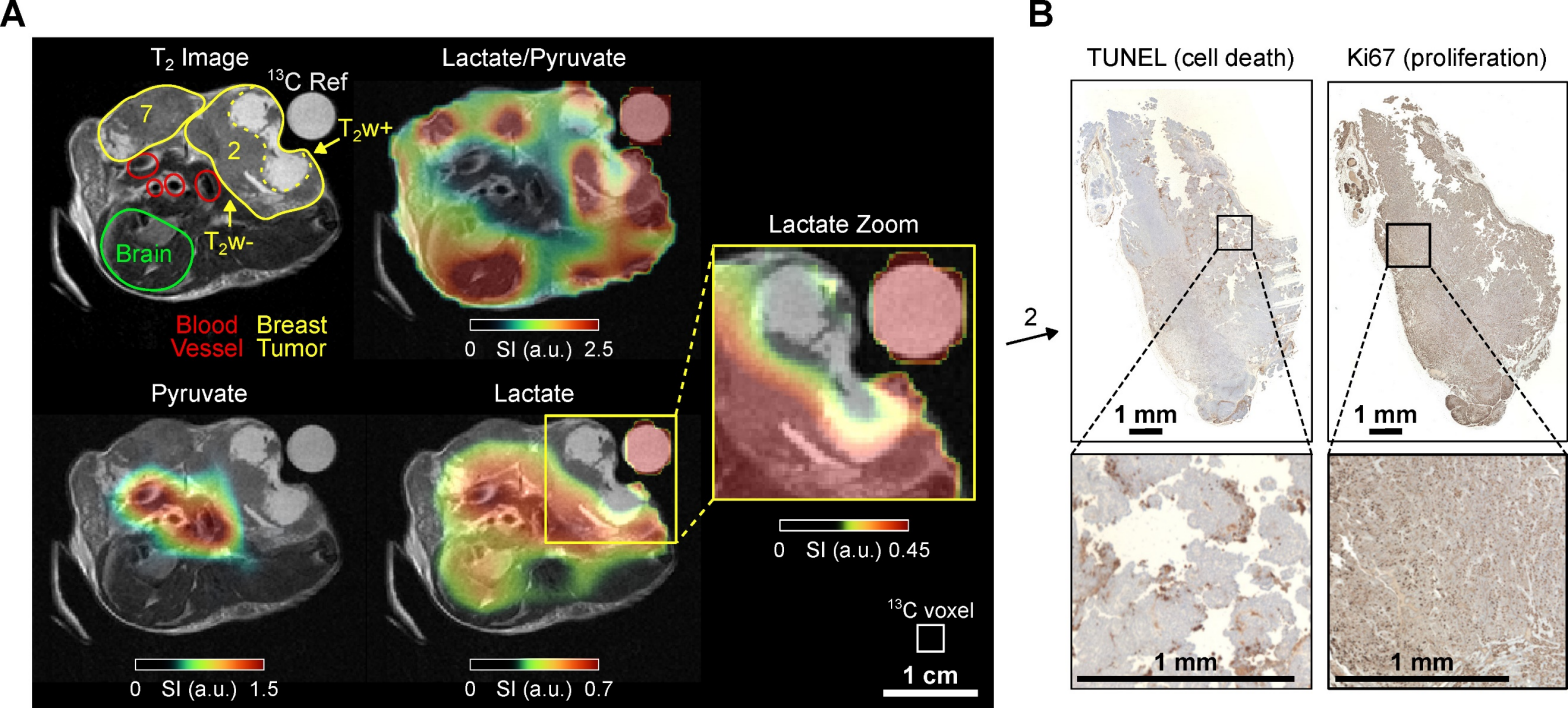
*In vivo* ^1^H T_2_w MRI and dynamic ^13^C chemical shift imaging of PyMT mouse C after administering SABRE-hyperpolarized [1-^13^C]-pyruvate-*d*_3_ and histology of the imaged tumor. (**A**) Axial anatomic ^1^H MRI showing the tumors on mammary glands 2 and 7, brain, blood vessels, and a ^13^C-lactate reference (left). The anatomic slice is superimposed with time-summated and interpolated ^13^C-lactate/^13^C-pyruvate (top right), ^13^C-pyruvate (bottom left) and ^13^C-lactate signal (bottom right). The heterogeneous breast tumor in mammary gland 2 divides into a hyper-intense (T_2_w+) region with lower lactate and lactate-to-pyruvate signals and a hypo-intense (T_2_w-) tumor region with higher signal. Note that low lactate signals can lead to high lactate-to-pyruvate ratios without significance if the pyruvate signal in the same voxel is negligible (no thresholding was applied to the ratio maps). The white box indicates the native CSI-FID resolution. (**B**) Histology of the hypointense tumor region with TUNEL staining indicating ongoing apoptosis (left) and Ki67 staining indicating cell proliferation (right).

Anatomical ^1^H MRI delineated the tumorous regions, revealing that the tumor in mammary gland 2 had progressed significantly, with a maximum diameter of 13 mm and notable T_2_w+ areas, **Figure 4**. This facilitated comparison with previous observations from mouse B, **Figure 3**. In the HP ^13^C MRI scan, consistent with previous results, robust pyruvate and lactate signals were detected in the tumors. Interestingly, the T_2_w+ region again exhibited lower metabolic activity than the surrounding tumor tissue, **Figure 4**. Histological analysis indicated apoptosis onset in the latter region. Note that the elevated lactate-to-pyruvate ratio in the lower right region probably corresponds to an extension of tumor 2. This area additionally shows a low SNR, particularly for the pyruvate signal (see supplementary **Figure S7G**).

### Summary of other mice and comparison

Three additional mice, D, E, and F, aged 12, 14, and 14 weeks respectively, were examined as detailed in the supplementary information (SI). They also exhibited localized tumor formations, consistent with observations from previous experiments, characterized by elevated ^13^C signals and lactate metabolism in tumor areas compared to healthy tissue, e.g., **Figure S9 and S10**. As previously described, T_2_w+ and necrotic regions showed meager ^13^C signals, i.e. were metabolically less active, **Figure S8**. In some cases, the tumors exhibited poor pyruvate signals but strong lactate signals, indicating rapidly metabolized pyruvate (mice B and C, **Figure S8)**.

**Figure 5:**
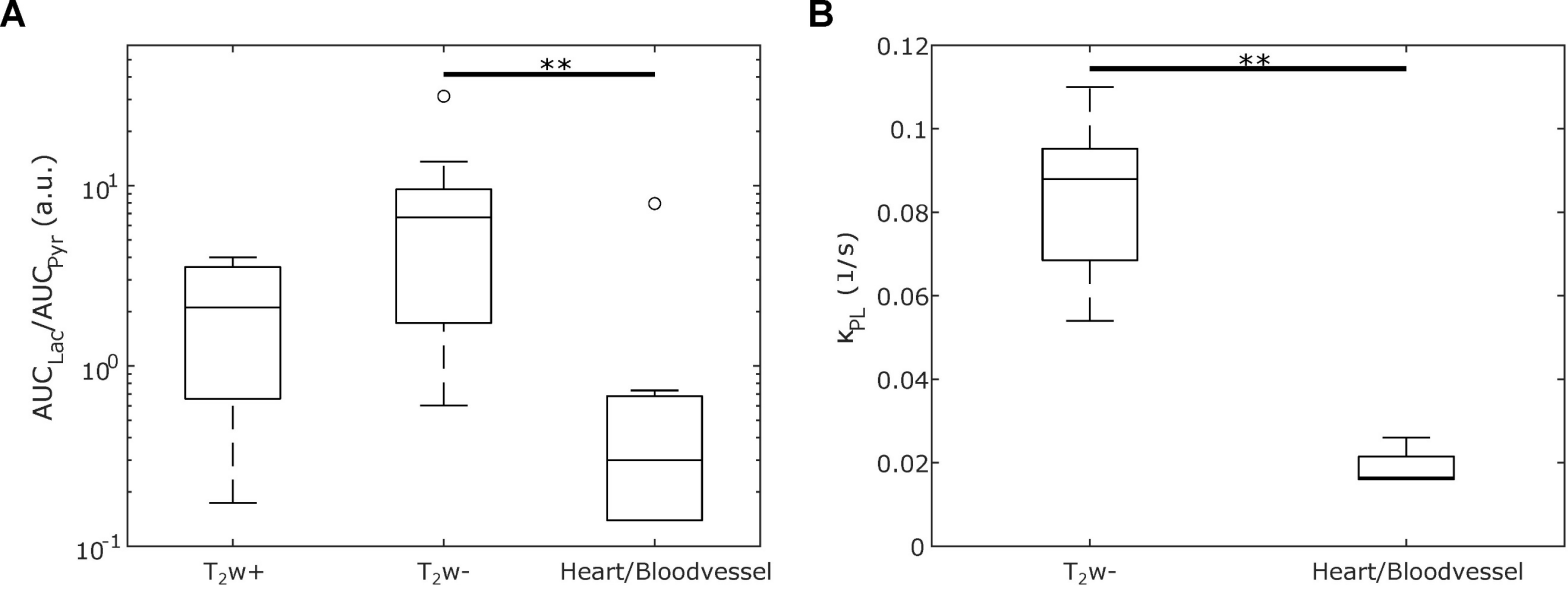
Box-and-whiskers plot depicting (**A**) the spectral lactate-to-pyruvate ratios and (**B**) the kinetic lactate-to-pyruvate rate constant *k*_PL_ across different regions in six mice: Hyperintense tumor (T_2_w+), hypointense tumor (T_2_w-), and heart resp. blood vessel (depending on the mouse). The significant difference between the T_2_w- and heart/blood vessel regions is marked with ** (p < 0.01).

The lactate-to-pyruvate ratios of different regions (T_2_w+, T_2_w-, and heart/blood vessel) from all six mice are compared in **Figure 5A**. T_2_w- tumor regions exhibit a higher lactate-to-pyruvate ratio compared to T_2_w+ tumor or heart/blood vessel regions, with a significant difference observed between T_2_w- and heart/blood vessel tissues (*p* = 0.0052). Pyruvate-to-lactate rate constants *k*_PL_ were derived from a kinetic model fitted to the dynamic data. A comparison of *k*_PL_ in T_2_w- tumor and heart/blood vessel regions is presented in **Figure 5B**, revealing a significant difference (p = 0.0060). The weighted averages of the *k*_PL_ in those regions are *k*_PL,T2w-_ = 0.081(13) 1/s, *k*_PL,Heart/Vessel_ = 0.0234(17) 1/s. Notably, a previous publication investigated the same breast cancer mouse model and supports the *k*_PL_ elevation compared to healthy tissue 15,52. Both trends, higher lactate-to-pyruvate ratios and elevated *k*_PL_ ​values in T_2_w- tumor regions, were observed across all mice, as shown in **Figures S11 and S12**.

## Significance and Conclusion

Breast cancer is one of the most prevalent tumor entities and has been studied extensively with dDNP-based HP MRI 53,54. Also, the MMTV-PyMT mouse model has been studied before 15. In this pilot study, we applied the less complex, low-cost, high-throughput SABRE approach for the first time to tumor imaging. Our methodology enables rapid preparation of HP pyruvate without chemical modification for *in vivo* use within six minutes, utilizing a cost-effective (<10,000 €), self-constructed setup.

In agreement with previous studies, we found elevated lactate formation as a manifestation of the Warburg effect in the tumors. Notably, we identified regional differences in pyruvate metabolism within the tumors in the ^13^C MRI. These findings correlated with histological results, particularly with cell proliferation and apoptosis staining.

We utilized transgenic MMTV-PyMT breast cancer mice 15,50,51. Tumor growth was fast, and substantial variations were observed between the six mice investigated. Via histology, we observed cell death onset within some tumors as the animals approached the ethical study endpoint regarding the primary tumor burden. Although this diversity in tumor stages complicates potential studies, the model is particularly intriguing because the mice hyper-activate key molecular pathways associated with breast cancer, such as PI3K signaling. The resulting stepwise tumor progression mirrors the tumor histopathology as well as the tumor stages of breast cancer patients. Therefore, the primary tumors of the MMTV-PyMT model present a range of metabolic tumor phenotypes that are likely to be encountered in clinical imaging studies. Notably, the clinical value of HP MRI has already been demonstrated in multiple clinical studies, including improved grading 9 and risk stratification for prostate cancer patients and characterization of tumor heterogeneity in glioblastoma 55. Recent reports showed the capability of HP MRI to monitor treatment response to neoadjuvant chemotherapy in breast cancer patients within days 11. Thus, the development of faster, cost-effective technologies is crucial for increasing the availability of this promising molecular imaging technique 53.

SABRE aims to address these issues. However, while the polarization levels currently achieved with SLIC-SABRE are sufficient for preclinical studies, they remain lower than those typically obtained with dDNP. This discrepancy limits its immediate applicability in settings requiring ultra-high signal-to-noise ratios. Similarly, PHIP has made significant advancements in recent years, achieving polarization levels comparable to dDNP. Yet, SABRE offers unique advantages, including a broader range of hyperpolarizable molecules and the absence of a chemical reaction shortly before injection. A key challenge for SABRE, however, are the short T_1_ relaxation times of some substrates during polarization build-up, which constrains the achievable polarization levels. It is worth mentioning that both dDNP and PHIP polarizers are already commercially available for preclinical applications.

Despite its advantages, translating SABRE to clinical applications will require significant effort, such as upscaling and enhanced purification of the contrast agent solutions. While our method, i.e. catalyst precipitation and methanol evaporation, currently reduces toxicity to acceptable levels in preclinical studies, further development of automated and scalable purification protocols is needed to ensure clinical safety. The currently remaining quantities of methanol and iridium are about one order of magnitude higher than permitted for clinical injection solutions 48.

While this pilot study provides a valuable demonstration, further research is required to build on these findings. Additional control studies are envisioned - such as imaging younger mice before apoptosis onset, longitudinal animal monitoring, and incorporating direct perfusion measurements - to provide deeper insights into the progression of tumors and the evolution of metabolic alterations, particularly the lactate-to-pyruvate ratio. These insights may help define biomarker thresholds indicative of tumor onset.

In conclusion, the present study demonstrates, for the first time, metabolic imaging of tumors with SLIC-SABRE hyperpolarized pyruvate. We have demonstrated that SABRE delivers the signal enhancement needed for detecting metabolic spatial differences in complex tumor tissue, possibly linked to different grades of proliferation and apoptosis. Note that with improved sequences, we also expect enhanced SNR and higher-quality images 56,57. To improve spatial accuracy and coregistration of regions of interest and mitigate partial volume effects, super-resolution techniques, such as patch-based reconstruction or volumetric super-resolution, may offer a viable approach to effectively reduce the thickness of the ^13^C slice 58. This study demonstrates that SABRE has matured enough to allow cancer studies, significantly enhancing access to HP as a powerful molecular imaging tool and being a promising candidate for clinical translation.

## Supplementary Material

Supplementary additional experimental details, MRI, and mice data.

## Abbreviations

HP: hyperpolarization/hyperpolarized
MRI: magnetic resonance imaging
TCA: tricarboxylic acid
dDNP: dissolution dynamic nuclear polarization
PHIP: parahydrogen-induced polarization
PHIP-SAH: parahydrogen-induced polarization by side-arm hydrogenation
SABRE: signal amplification by reversible exchange
SLIC-SABRE: spin-lock induced crossing signal amplification by reversible exchange
PBS: phosphate-buffered solution
MMTV-PyMT: mouse mammary tumor virus - polyomavirus middle tumor-antigen
PI3K: phosphoinositide 3-kinase
FID-CSI: free-induction decay chemical shift imaging
ROI: region of interest
T_2_w+: T_2_ hyper-intense
T_2_w-: T_2_ hypo-intense
TUNEL: terminal deoxynucleotidyl transferase dUTP nick end labeling
