# Supplementary material for: In vivo molecular imaging of breast cancer metabolic heterogeneity using [1-13C]pyruvate-d3 hyperpolarized by reversible exchange with parahydrogen
Source: Theranostics. 2025 Mar 3;15(9):3714–23. doi: 10.7150/thno.103272 (PMC11980672; doi:10.7150/thno.103272)
Supplement: Supplementary file 1 — Supplementary additional experimental details, MRI, and mice data. [file thnov15p3714s1.pdf]

Supporting Information

In vivo molecular imaging of breast cancer metabolic heterogeneity using [1-^{13}C]pyruvate-d_3 hyperpolarized by reversible exchange with parahydrogen

Stefan Petersen,[‡] *Luca Nagel,*[‡] *Philipp R. Groß, Henri de Maissin, Robert Willing, Lisa Heß, Julia Mitschke, Nicole Klemm, Judith Treiber, Christoph A. Müller, Stephan Knecht, Ilai Schwartz, Moritz Weigt, Michael Bock, Dominik von Elverfeldt, Maxim Zaitsev, Eduard Y. Chekmenev, Jan-Bernd Hövener, André F. Martins, Franz Schilling, Thomas Reinheckel, and Andreas B. Schmidt**

[‡] shared first authorship

Table of Contents

Experimental Procedures 2
- Sample Preparation 2
- Experimental Setup 2
- Hyperpolarization and Purification 3

Mouse Model 4

In Vivo Experiments 4

MRI Experiments 5
- MRI Sequences 5
- Processing 5
- Mouse A (ID 5441) 7
- Mouse B (ID 5366) 9
- Mouse C (ID 5449) 10
- Mouse D (ID 5362) 11
- Mouse E (ID 5383) 12
- Mouse F (ID 5389) 13
- Lactate-to-Pyruvate Ratios and Kinetic Rate Constants 14
- Non-interpolated images 17

Histology of tumors 18
- H&E staining 18
- TUNEL staining for visualization of cell death 18
- Immunohistochemistry 18
- Vascularization/CD31 stain images 19
- Histological Staining Quantification 20

References 21

Experimental Procedures

Sample Preparation

The samples were prepared using 50 mM sodium [1-^{13}C]-pyruvate-d$_3$, 6 mM IrIMes(COD)Cl pre-catalyst, 30 mM DMSO-d_6 (Sigma Aldrich) and 0.65 mM ethylenediaminetetraacetic acid (EDTA) in 600 µl methanol-d_4. The methanol-d_4 was degassed before mixing by introducing N_2 gas into the solution for 2 min. After mixing, the samples were sonicated for ≈10 min and passed through a 1.2 µm syringe filter.

The IrIMes(COD)Cl pre-catalyst was synthesized using an established procedure [1].

Sodium [1-^{13}C]-pyruvate-d_3 was formed by reacting [1-^{13}C]-pyruvic acid-d_4 (catalogue number 900845-SPEC, Sigma Aldrich, ≥99 atom % ^{13}C, ≥97 atom % D) with NaOD (sodium deuteroxide solution, 40 wt. % in D_2O, CAS: 14014-06-3, Sigma, 99 atom % D). This reaction took place in a flask filled with D_2O, which was placed in an ice water bath. Any remaining D_2O was removed in a lyophilizer freeze dryer to eliminate moisture.

The phosphate buffered solutions (PBS) added for the purification were prepared by dissolving commercially purchased phosphate buffer powder (pH 7.3-7.5, 0.017 g/mL in water, high purity, SKU P7994-1EA, Sigma Aldrich) in deionized D_2O.

The parahydrogen (≈90% enrichment) used for the hyperpolarization was produced in a previously described setup [2].

Experimental Setup

The hyperpolarization was performed in a custom-built setup as described in our previous paper [3]. The static magnetic field along the z-axis (B_0) was generated using a resistive solenoid coil (360 mm length, 120 mm inner diameter, two layers of 1.12 mm copper wire, 47 compensation windings at each end of the solenoid). The B_1 field along the x-axis for SLIC polarization transfer was generated using a saddle-shaped coil (one winding around a 320 mm long, 100 mm inner diameter tube) and a custom-written Python script controlling the analog output of a digital-to-analog converter (DAC, NI USB-6251, National Instruments, USA). Before being transmitted to the B_1 coil, the pulses were amplified using a 12-Watt audio amplifier (KEMO MO32S). The external fields were shielded using a three-layer mu-metal (ZG-209, Magnetic Shield Corp.). The B_0 and B_1 deviated by ± 1% over the sample volume (measured with a Fluxgate-Magnetometer Fluxmaster, Stefan-Mayer Instruments Germany). The hyperpolarization was performed in 5-mm medium-wall NMR tubes (Wilmad Labglass) using a home-built bubbling setup based on published designs [4–6] and a water bath regulating the sample temperature.

The NMR measurements for evaluating the hyperpolarization values were performed in a 1 T benchtop NMR system (Spinsolve Carbon 43, Magritek). The MRI experiments were performed using a 7 T preclinical MRI system (Biospec 70/20, PV6.0.1, Bruker, Germany) with a proton-carbon quadrature-quadrature coil (V-XLS-HL-070-01349 V01, Rapid, Germany). The MRI sequences and protocols are described in the SI section "MRI Experiments".

Hyperpolarization and Purification

The hyperpolarization and purification process were performed according to our recently published protocol [3]: We hyperpolarized 50 mM [1-^{13}C]-pyruvate-d_3 to up to 24% in the presence of 6 mM Iridium(COD)IMes catalyst and 30 mM DMSO-d_6 in 600 µL CD_3OD using spin-lock induced crossing (SLIC-) SABRE [7] at a 50 µT static magnetic field. Subsequently, we added 600 µL pH-buffered D_2O and evaporated methanol-d_4 at approximately 100 mT magnetic field, 5mbar pressure and 100 °C temperature. The solution was then filtered to remove precipitated iridium catalyst in the aqueous phase, extracting the 50 mM [1-^{13}C]-pyruvate-d_3 in a D_2O solution of pH 6.5 – 7, ready for administration to the mice (residual 250 mM methanol, 20 µM catalyst), **Figure 1**. This way, we efficiently produced a batch of ≈ 250 µL containing ≈30 mM aqueous HP [1-^{13}C]-pyruvate-d_3, ^{13}C-hyperpolarized up to $P_{13C} \approx 10\%$ in less than 6 minutes, **Figure S1**.

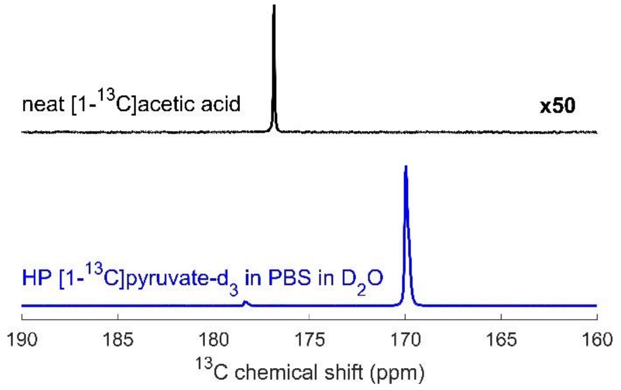

Figure S1. ^{13}C NMR spectra of hyperpolarized [1-^{13}C]-pyruvate-d_3 in PBS in D_2O after purification (blue) and a thermally polarized reference solution containing 17.4 M [1-^{13}C]-acetic acid (black) at 1 T.

The ^{13}C polarization was calculated by using a thermally polarized external ^{13}C reference sample (neat [1-^{13}C]acetic acid, CAS: 1563-79-7, 99 atom % ^{13}C, Sigma Aldrich) using following equation:

$$P_{hyp} = \frac{I_{hyp}}{I_{ref}} \cdot \frac{c_{ref}}{c_{hyp}} \cdot \frac{N_{ref}}{N_{hyp}} \cdot \frac{f_{ref}}{f_{hyp}} \cdot P_{ref}$$

I denotes the integrated signal, *c* the concentration, *f* the ^{13}C enrichment, and *N* the number of averages (for both measurements N = 1) of the hyperpolarized "hyp" or reference sample "ref". P_{ref} is the thermal polarization of the reference sample at room temperature (298 K) and 1 T magnetic field of the NMR system.

Mouse Model

For the described in vivo experiments, the MMTV-PyMT mouse model of metastasizing breast cancer was used . In this mouse model, the Polyoma Middle T antigen is expressed in the mammary tissue leading to the development of breast tumors by hyperactive phosphoinositide 3-kinase (PI3K) pathway. Tumor onset is mostly at around 6 weeks of age reaching carcinoma state at about 14 weeks of age. All ten mammary glands numbered in Figure S2 potentially develop breast tumors, but with a high heterogeneity concerning structure of the tumor and the speed of development .

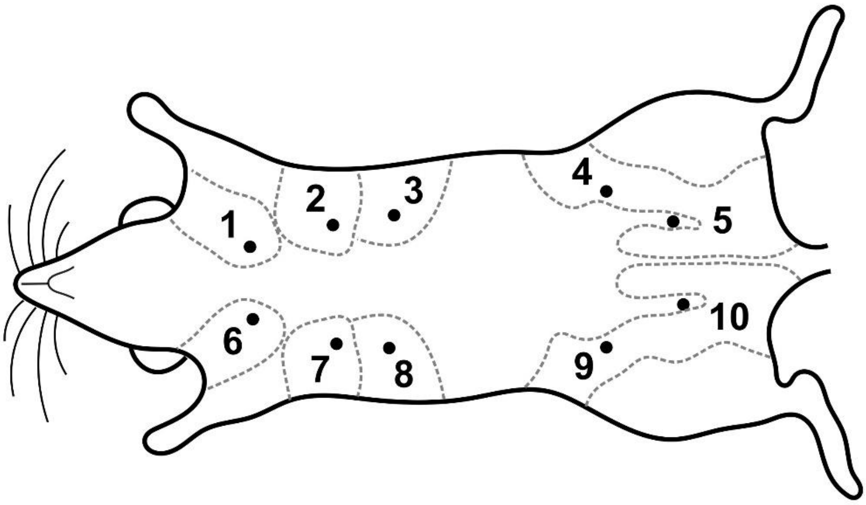

Figure S2. Illustration of a mouse with mammary glands numbered as used in this study. The corresponding tumors are denoted by the same numbering system.

In Vivo Experiments

Ethical approval for the animal experiments was obtained from the relevant authority (Regierungspraesidium Freiburg, Talstr. 4-8, 79095 Freiburg; AZ: 35-9185.81/G-21/134). Anesthesia was induced using isofluorane (1-4% in >99.5% O_2, ≈1.0 L·min^{-1}, during spontaneous breathing), and vital signs were continuously monitored (SA Instruments 1030, Stony Brook, NY 11790). Respiratory rate was maintained at approximately 70 min^{-1}, with anesthesia depth adjusted as necessary. Body temperature was monitored using a rectal thermometer and stabilized using a custom-made water circulation system driven by a water pump. All necessary measures were taken to minimize suffering, and humane euthanasia was performed at the end of the experiment. The bolus of 30 mM HP [1-^{13}C]-pyruvate-d_3 solution was administered within ≈8 s through the tail vein (≤5 µL/g mouse weight).

MRI Experiments

MRI Sequences

^{13}C MRI were acquired using 2D FID chemical shift imaging (FIDCSI) and ^{1}H T_2w RARE MRI were acquired for anatomical reference. The detailed MRI sequences and protocols are described in the following subsections along with the measured mice. Note that ^{13}C MRI data of mice B and D were acquired statically starting 10s and 20s post-injection, respectively, while the other mice were acquired using a dynamic ^{13}C CSI.

Processing

Pyruvate and lactate images were generated by time domain fitting using Python 3.10. This was done by iteratively minimizing the sum-of-squares R^2 difference between the measured data $S(t)$ and a modeled Free Induction Decay FID $F(t)$ while adjusting the models amplitude A_n, T_{2n}* and frequency ω_n for N=4 metabolites pyruvate, lactate, alanine, pyruvate hydrate:

$$F(t) = \sum_{n=0}^{N=3} A_n \cdot exp(i\,\omega_n\,t) \cdot exp(-t/T_{2n}^*)$$

$$R^2 = \sum_{t=0}^{T} (|F(t) - S(t)|^2)$$

In case of dynamic CSI, the fitted FID amplitudes A_n were averaged along the time domain (repetitions) before taking the absolute values of the mean amplitudes. In case of single-repetition acquisitions, the absolute value of amplitude was taken. These values were then used for the pyruvate and lactate maps.

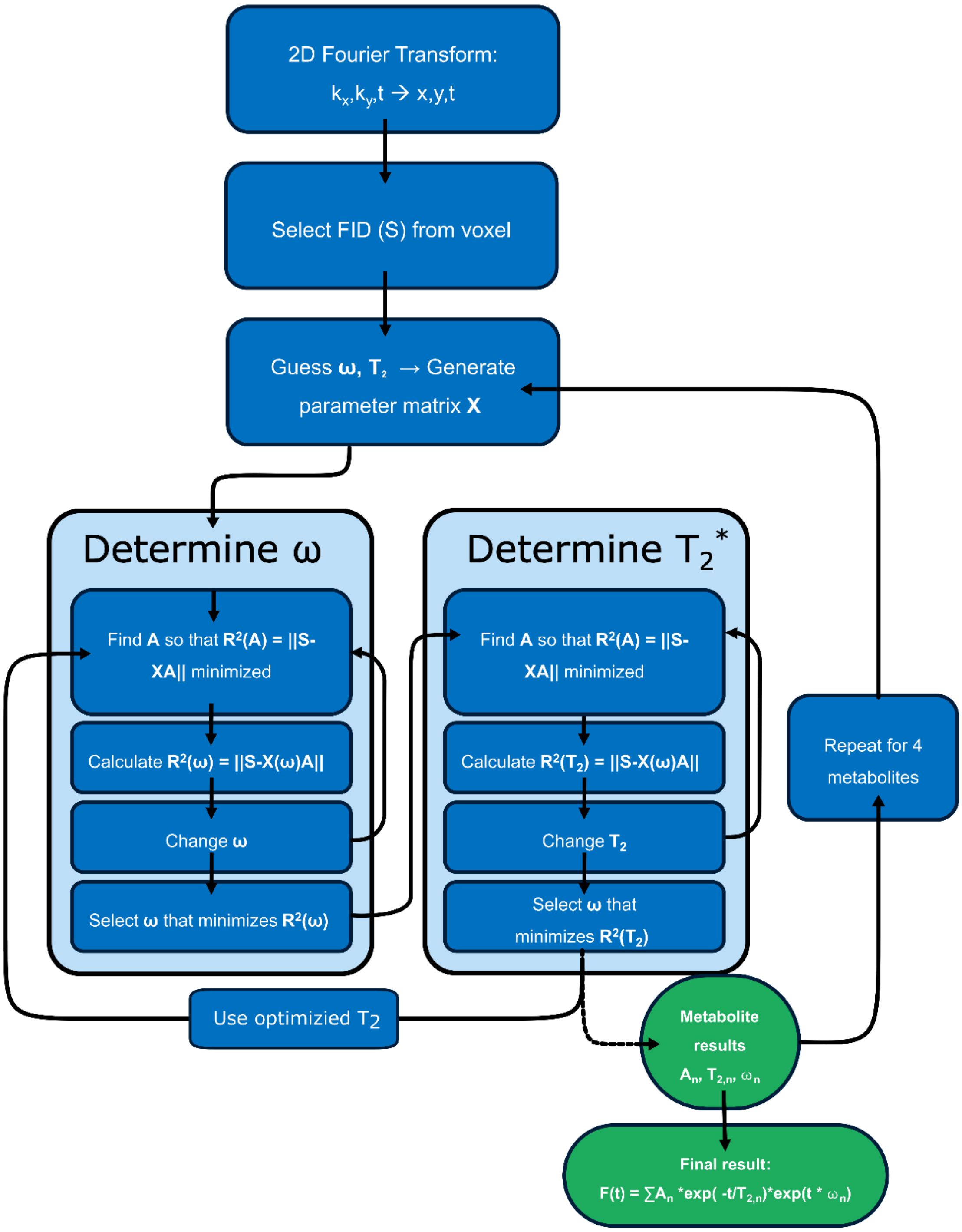

Figure S3. The figure shows the processing pipeline for the CSI datasets. After a 2D Fourier Transform (k_x,k_y,t) → (x,y,t), FIDs $S(t)$ from each voxel are processed individually. The inital parameters (ω, T_2*) are set. Then the best matching amplitude A is determined, and ω is incremented. This procedure is repeated and each time the difference R between the measured signal $S(t)$ and the modelled signal XA is determined. After the optimal frequency ω is found, the same process is repeated with T_2* and after that the minimization is repeated for the other metabolites. The whole process is repeated several times until optimal parameters can be extracted.

To extract the ROI spectra, each voxel was weighted by the percentage of its volume that fell within the ROI. The ROI spectra were then calculated as a weighted average of the magnitude spectra of these voxels.

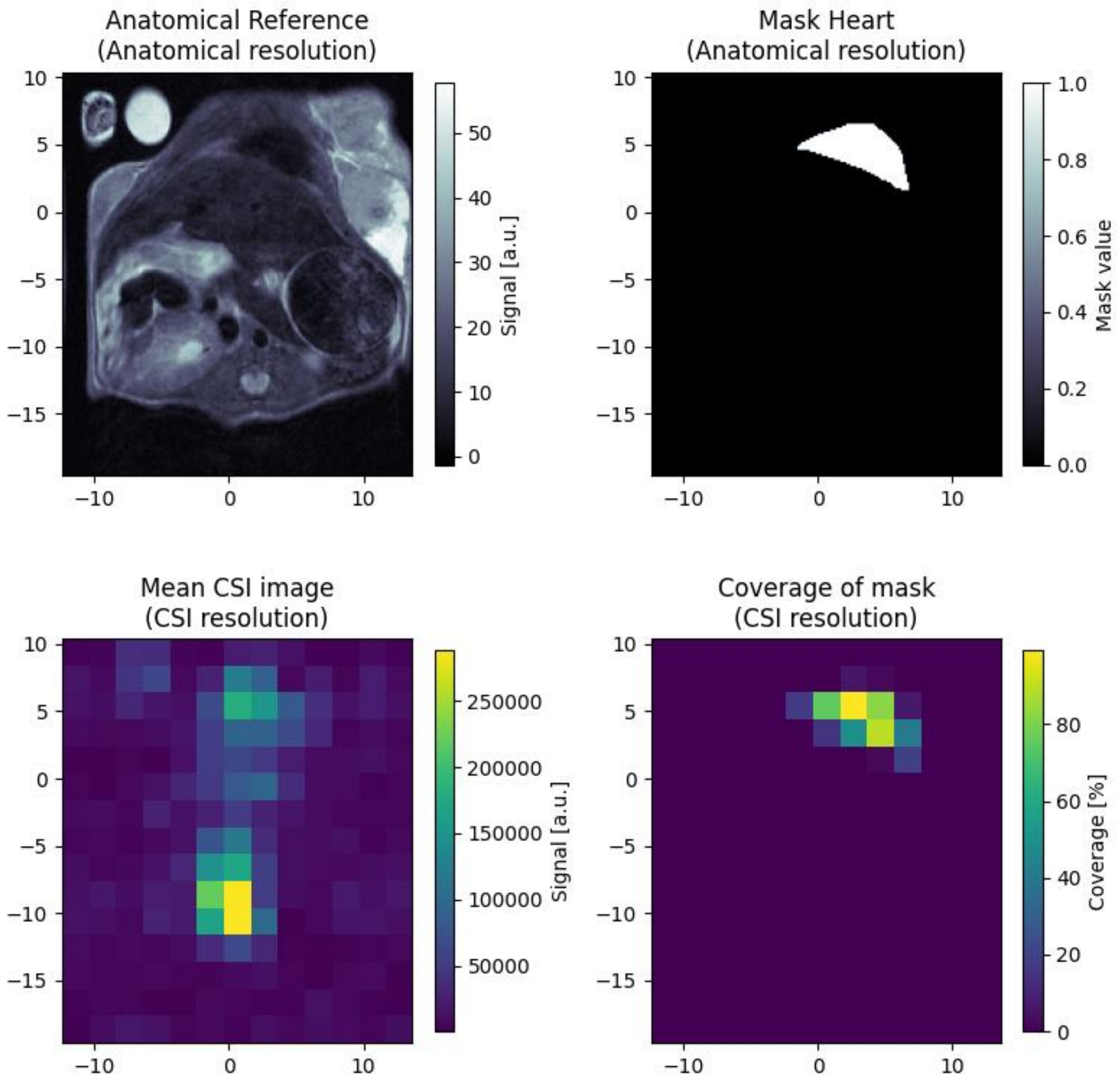

Figure S4. Figure depicting the masking process to generate weighted spectra. High resolution masks (upper right) are drawn onto high resolution anatomical images (upper left). The masks are then converted into the lower resolution of the CSI image (lower left), where each voxel contains information of the coverage (lower right) of the high resolution mask of the low resolution CSI image.

The metabolite maps were interpolated to one of the anatomical ^{1}H image using the MATLAB function ‘imresize’ with bilinear interpolation and normalized to a saturation value. A transparency cut-off was added (as depicted in black in the color bars in the shown ^{13}C MRI images) to achieve optimal visualization of both anatomy and metabolism.

Mouse A (ID 5441)

Mouse: ≈13 weeks old, m = 24 g, T = 37.3 °C.

^{13}C MRI: Dynamic 2D FID chemical shift imaging (FIDCSI), FOV: 24x30 mm^2; matrix: 8x10; slice thickness: 5 mm; spectral bandwidth 2000 Hz (80 points); T_R = 43.2 ms; α = 5°; N = 1; N_R = 40; time resolution: 3.46 s.

^{1}H MRI: Axial T$_2$w multi-slice RARE MRI, RARE-factor with fat saturation on: 12, FOV: 27x27 mm^2, matrix: 200x200, slice thickness: 0.8 mm; T_R = 4 s; T_E = 6.3 ms; α = 90°, N = 4.

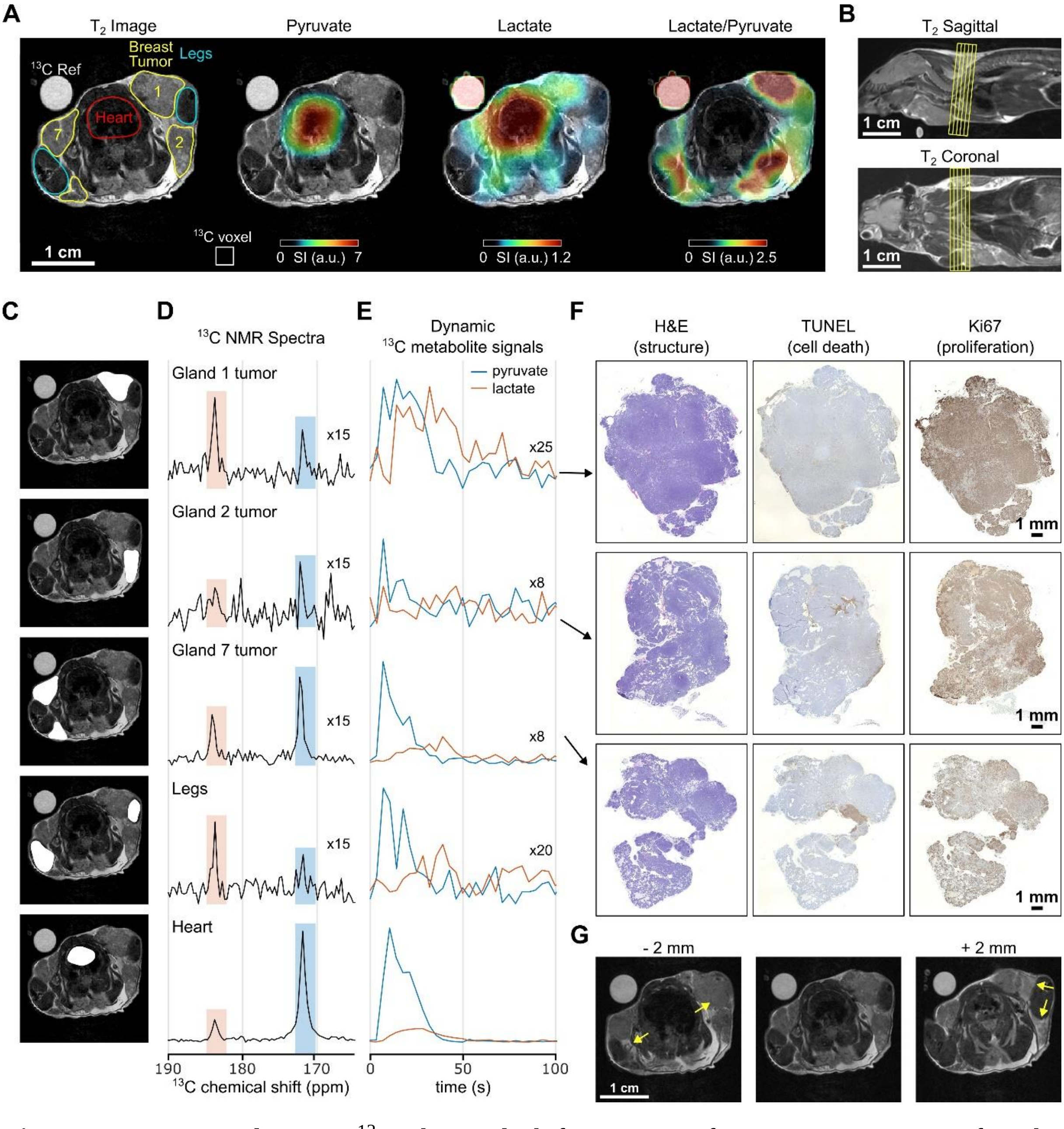

Figure S5. In vivo dynamic ^{13}C chemical shift imaging of a PyMT mouse A after the administration of SABRE-hyperpolarized [1-^{13}C]-pyruvate-d$_3$ and histology of the imaged tumors. **(A)** ^{1}H MRI showing an axial anatomic slice used for metabolic imaging, superimposed with time-summated and interpolated ^{13}C-pyruvate, ^{13}C-lactate, and ^{13}C-lactate/^{13}C-pyruvate signal. The original resolution is indicated by a white box. (**B**) The ^{13}C slice orientation and thickness is indicated by yellow lines in the sagittal (top) and coronal (bottom) ^{1}H MRI. (**C**) Anatomical ^{1}H MRI showing regions of interests (ROI, white) used to calculate the voxel normalized ^{13}C NMR spectra (**D**) and dynamic ^{13}C metabolite signals (**E**): Tumor on gland 1, 2 and 7, legs, and heart (top to bottom). (**F**) Histology of the breast tumors with different staining indicating the structure (H&E, left), apoptosis (TUNEL, center), and cell proliferation (Ki67, right). **(G)** Axial ^{1}H-MRI slices shifted by -2mm (left) and +2mm (right) from the center slice (center), which was used for ROI definition. Arrows indicate tissue displacements relevant for the analysis of ^{13}C signal images.

Mouse B (ID 5366)

Mouse: 12 weeks old, m = 24 g.

^{13}C MRI: 2D FID chemical shift imaging (FIDCSI); FOV: 26x30mm^2; matrix: 13x15, slice thickness: 7 mm; spectral bandwidth 2500 Hz (200 points); T_R = 85 ms; α = 12°; N = 1. Image acquired 10 s post-injection.

^{1}H MRI: Axial T_2w multi-slice RARE MRI with fat saturation on; RARE-factor: 12; FOV: 26x30 mm^2; matrix: 260x300; slice thickness: 1 mm; T_R = 4 s; T_E = 5.007 ms; α = 90°; N = 9.

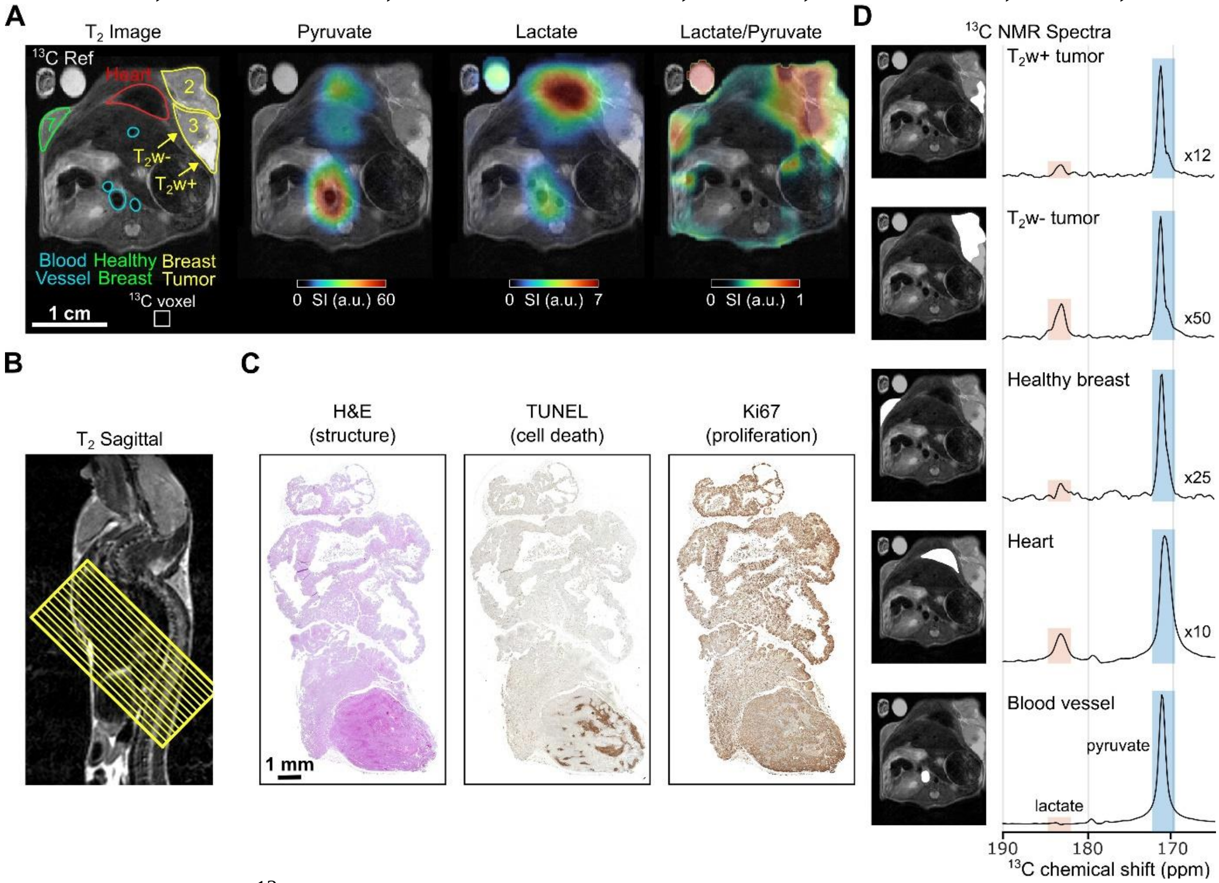

Figure S6. In vivo ^{13}C chemical shift imaging of a PyMT mouse B after the administration of SABRE-hyperpolarized [1-^{13}C]-pyruvate-d_3 and histology of the imaged tumor. (**A**) ^{1}H MRI showing an axial anatomic slice used for metabolic imaging, superimposed with the interpolated ^{13}C-pyruvate, ^{13}C-lactate, and ^{13}C-lactate/^{13}C-pyruvate signal. The original resolution is indicated by a white box. (**B**) The ^{13}C slice orientation and thickness is indicated by yellow lines in the sagittal ^{1}H MRI. (**C**) Histology of the breast tumor with different staining indicating the structure (H&E, left), apoptosis (TUNEL, center), and cell proliferation (Ki67, right). (**D**) Anatomical ^{1}H MRI (left) showing regions of interests (white) used to calculate the voxel normalized ^{13}C NMR spectra (right): Hyperintense tumor (T_2w+), hypointense tumor (T_2w-), healthy breast, heart, and blood vessel (top to bottom). The spectra were linebroadened with a Gaussian (FWHM 25Hz).

Mouse C (ID 5449)

Mouse: ≈13 weeks old, m = 26.7 g, T = 37.1 °C.

^{13}C MRI: Dynamic 2D FID chemical shift imaging (FIDCSI); FOV: 24x30 mm^2; matrix: 8x10; slice thickness: 5 mm; spectral bandwidth 2000 Hz (80 points); T_R = 43.2 ms; α = 5°; N = 1; N_R = 40; time resolution: 3.46 s.

^{1}H MRI: Axial T_2w multi-slice RARE MRI with fat saturation off; RARE-factor: 12; FOV: 24x30 mm^2; matrix: 160x200; slice thickness: 0.8 mm; T_R = 4 s; T_E = 6.3 ms; α = 90°; N = 4.

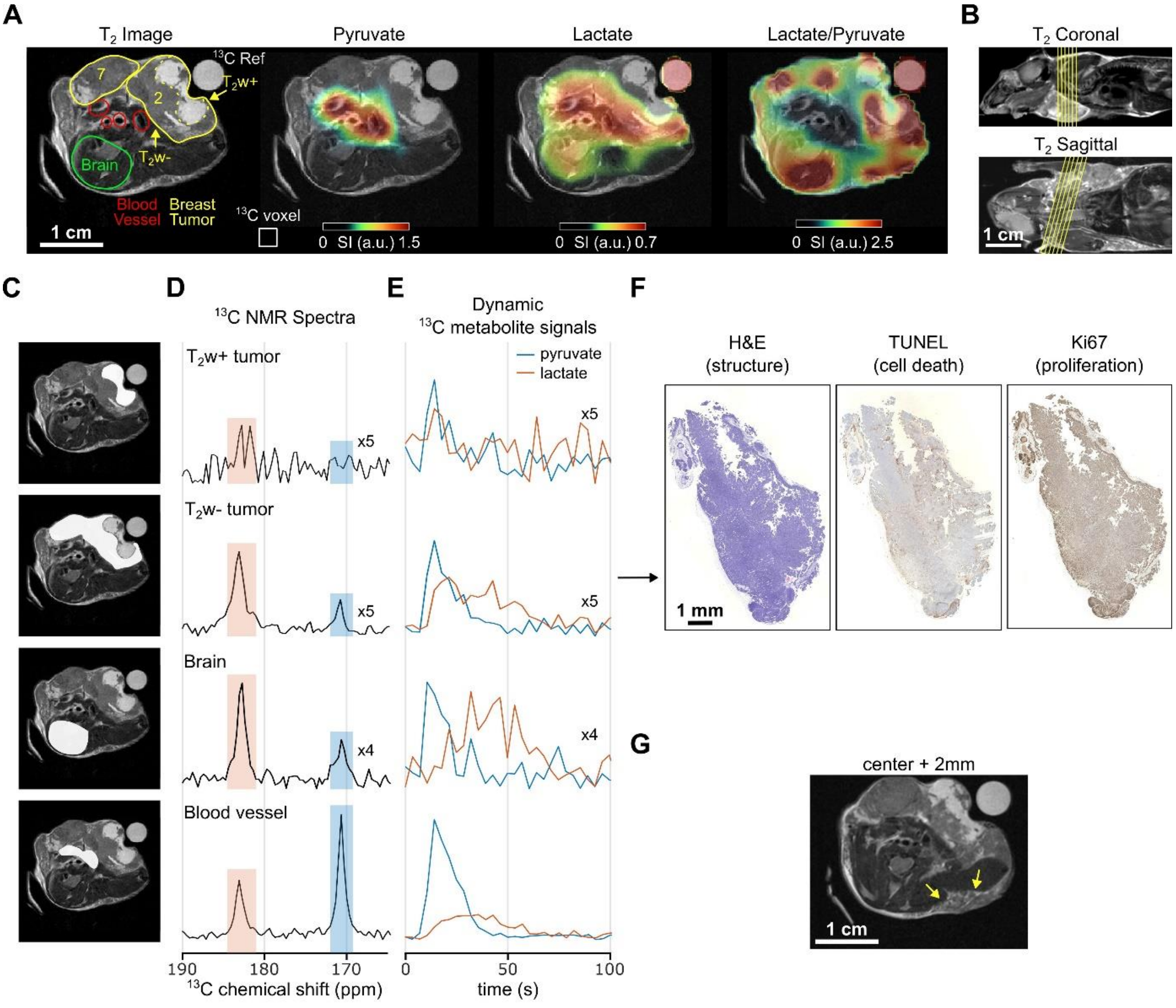

Figure S7. In vivo dynamic ^{13}C chemical shift imaging of a PyMT mouse C after the administration of SABRE-hyperpolarized [1-^{13}C]-pyruvate-d$_3$ and histology of the imaged tumor. **(A)** ^{1}H MRI showing an axial anatomic slice used for metabolic imaging, superimposed with time-summated and interpolated ^{13}C-pyruvate, ^{13}C-lactate, and ^{13}C-lactate/^{13}C-pyruvate signal. The original resolution is indicated by a white box. **(B)** The ^{13}C slice orientation and thickness is indicated by yellow lines in the sagittal (top) and coronal (bottom) ^{1}H MRI. **(C)** Anatomical ^{1}H MRI showing regions of interests (white) used to calculate the voxel normalized ^{13}C NMR spectra (**D**) and dynamic ^{13}C metabolite signals (**E**): Hyperintense tumor (T_2w+), hypointense tumor (T_2w-), and blood vessel (top to bottom). **(F)** Histology of the hypointense tumor with different staining indicating the structure (H&E, left), apoptosis (TUNEL, center), and proliferation (Ki67, right). **(G)** 2mm off-center axial anatomic slice showing an extension of tumor 2, as highlighted with arrows, possibly explaining the elevated Lac/Pyr ratio observed in this area.

Mouse D (ID 5362)

Mouse: 12 weeks old, m = 24 g.

^{13}C MRI: 2D FID chemical shift imaging (FIDCSI); FOV: 26x32mm^2; matrix: 13x16; slice thickness: 5 mm; spectral bandwidth 2500 Hz (200 points); T_R = 85 ms; α = 12°; N = 1. Image acquired 20 s post-injection.

^{1}H MRI: Axial T$_2$w multi-slice RARE MRI with fat saturation on; RARE-factor: 12; FOV: 26x32 mm^2; matrix: 173x213; slice thickness: 1 mm; T_R = 4 s; T_E = 4.0027 ms; α = 90°; N = 9.

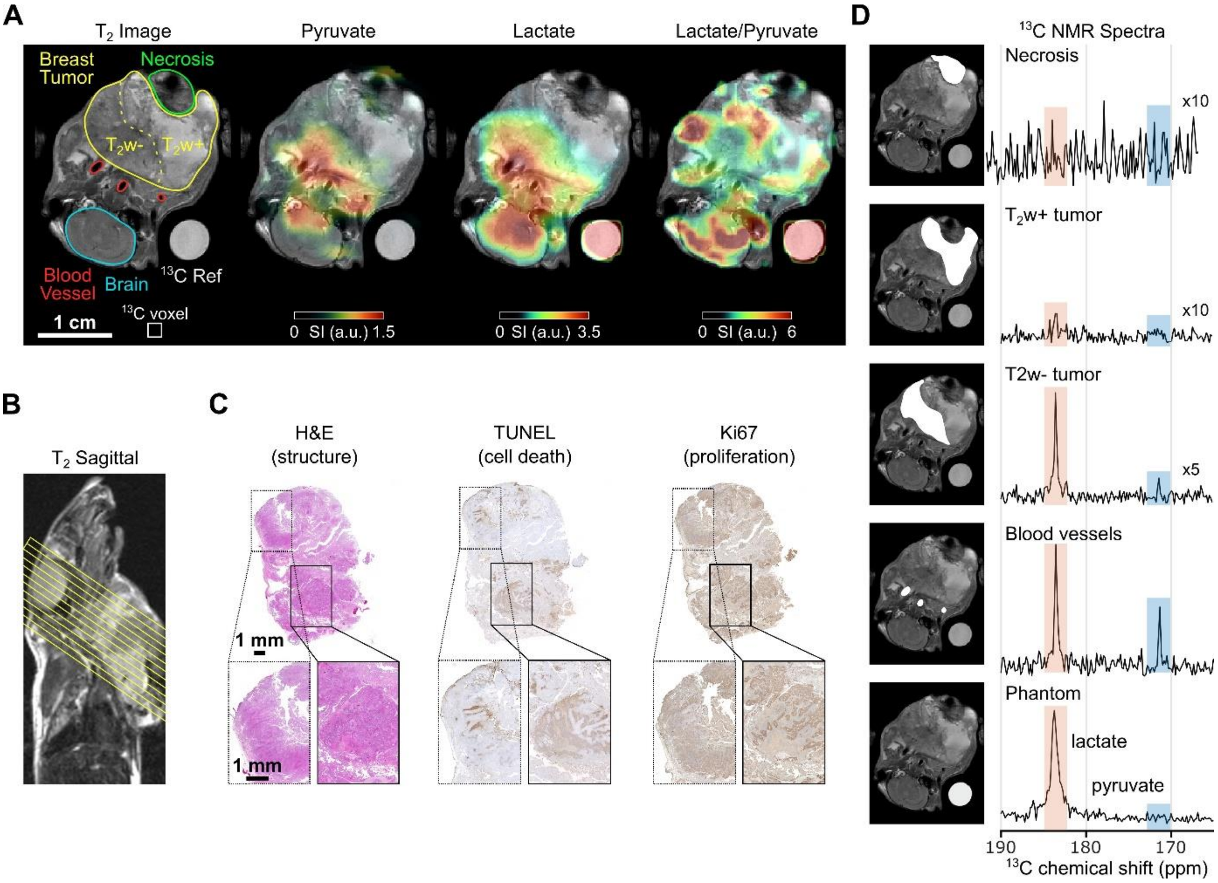

Figure S8. In vivo ^{13}C chemical shift imaging of a PyMT mouse D after the administration of SABRE-hyperpolarized [1-^{13}C]-pyruvate-d$_3$ and histology of the imaged tumors. (**A**) ^{1}H MRI showing an axial anatomic slice used for metabolic imaging, superimposed with the interpolated ^{13}C-pyruvate, ^{13}C-lactate, and ^{13}C-lactate/^{13}C-pyruvate signal. The original resolution is indicated by a white box. (**B**) The ^{13}C slice orientation and thickness is indicated by yellow lines in the sagittal ^{1}H MRI. (**C**) Histology of the breast tumor with different staining indicating the structure (H&E, left), apoptosis (TUNEL, center), and proliferation (Ki67, right). (**D**) Anatomical ^{1}H MRI (left) showing regions of interests (white) used to calculate the voxel normalized ^{13}C NMR spectra (right): Necrosis, hyperintense tumor (T$_2$w+), hypointense tumor (T$_2$w-), blood vessel, and phantom (top to bottom).

Mouse E (ID 5383)

Mouse: ≈14 weeks old, m = 28.4 g, T = 35.8 °C.

^{13}C MRI: Dynamic 2D FID chemical shift imaging (FIDCSI); FOV: 26x25mm^2; matrix: 10x10; slice thickness: 5 mm; spectral bandwidth 2000 Hz (80 points); T_R = 41.7 ms; α = 5°; N = 1; N_R = 40; time resolution: 4.17 s.

^{1}H MRI: Axial T_2w multi-slice RARE MRI with fat saturation off; RARE-factor: 12; FOV: 26x25 mm^2; matrix: 167x173; slice thickness: 1 mm; T_R = 5 s; T_E = 3.7 ms; α = 90°; N = 3.

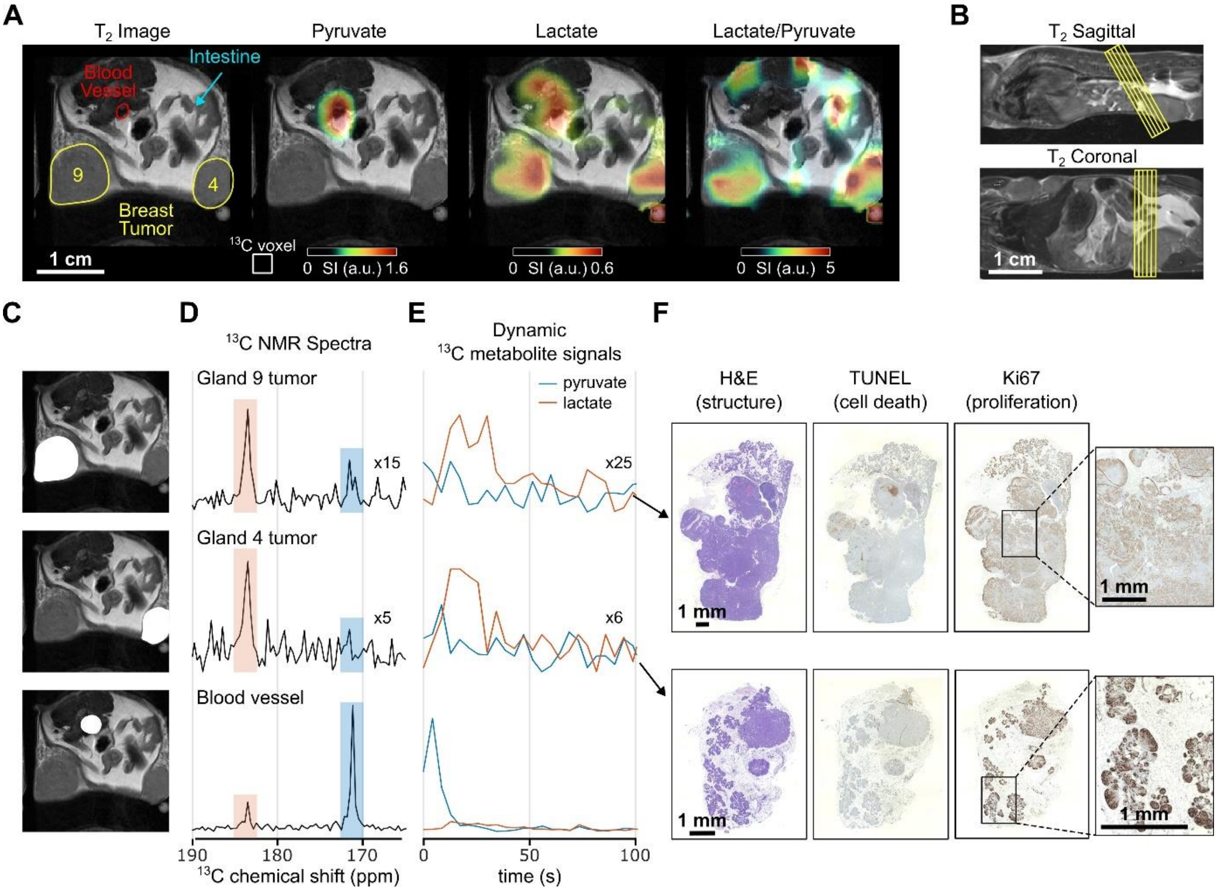

Figure S9. In vivo dynamic ^{13}C chemical shift imaging of a PyMT mouse A after the administration of SABRE-hyperpolarized [1-^{13}C]-pyruvate-d$_3$ and histology of the imaged tumors. **(A)** ^{1}H MRI showing an axial anatomic slice used for metabolic imaging, superimposed with time-summated and interpolated ^{13}C-pyruvate, ^{13}C-lactate, and ^{13}C-lactate/^{13}C-pyruvate signal. The original resolution is indicated by a white box. (**B**) The ^{13}C slice orientation and thickness is indicated by yellow lines in the sagittal (top) and coronal (bottom) ^{1}H MRI. **(C)** Anatomical ^{1}H MRI showing regions of interests (white) used to calculate the voxel normalized ^{13}C NMR spectra (**D**) and dynamic ^{13}C metabolite signals (**E**): Tumor on gland 9 and 4, and blood vessel (top to bottom). (**F**) Histology of the breast tumors with different staining indicating the structure (H&E, left), apoptosis (TUNEL, center), and proliferation (Ki67, right).

Mouse F (ID 5389)

Mouse: ≈14 weeks old, m = 25.4 g, T = 36.4 °C.

^{13}C MRI: Dynamic 2D FID chemical shift imaging (FIDCSI); FOV: 27x30 mm^2; matrix: 9x10; slice thickness: 5 mm; spectral bandwidth 2000 Hz (80 points); T_R = 42.3 ms; α = 5°; N = 1; N_R = 40; time resolution: 3.81 s.

^{1}H MRI: Axial T_2w multi-slice RARE MRI with fat saturation on; RARE-factor: 12; FOV: 27x30 mm^2; matrix: 180x200; slice thickness: 1 mm; T_R = 4 s; T_E = 3.8 ms; α = 90°; N = 10.

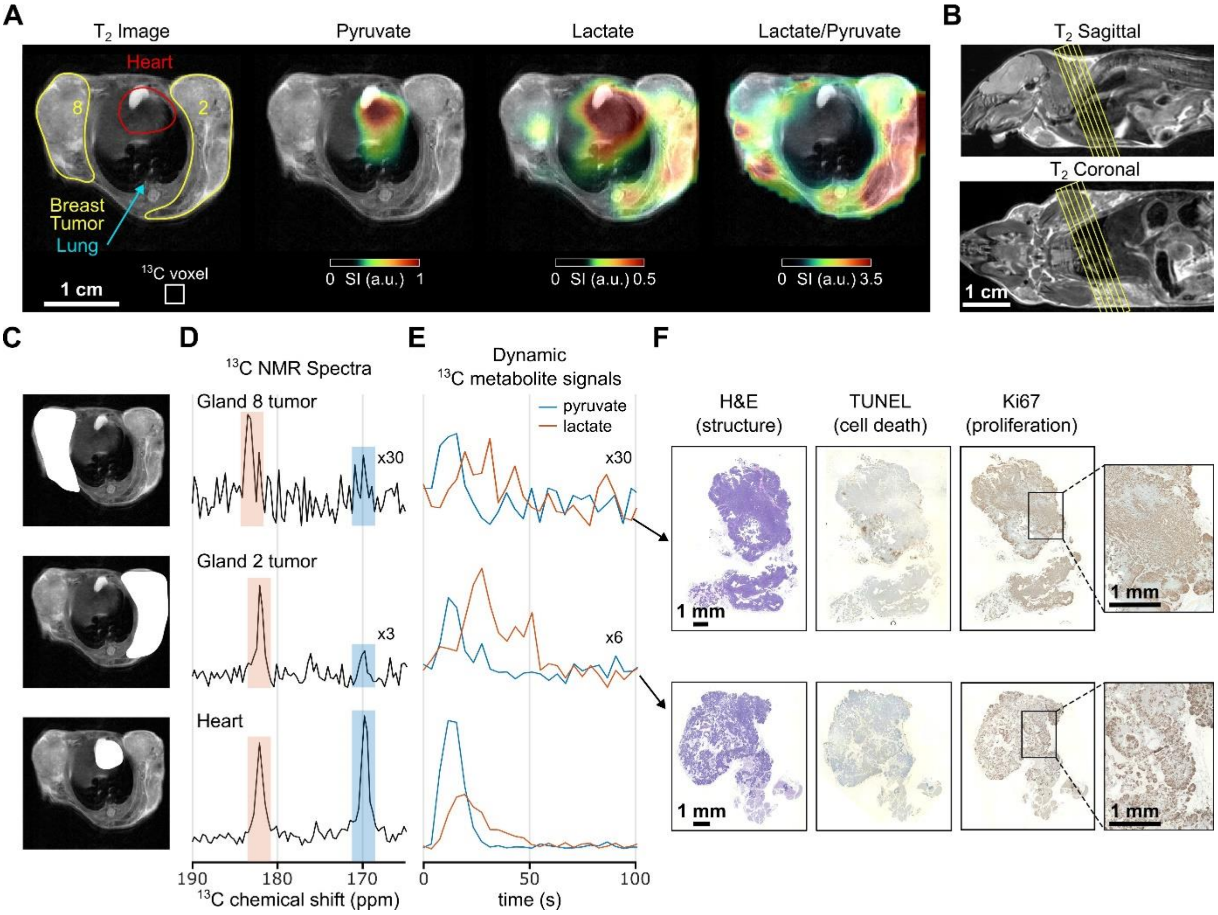

Figure S10. In vivo dynamic ^{13}C chemical shift imaging of a PyMT mouse A after the administration of SABRE-hyperpolarized [1-^{13}C]-pyruvate-d_3 and histology of the imaged tumors. **(A)** ^{1}H MRI showing an axial anatomic slice used for metabolic imaging, superimposed with time-summated and interpolated ^{13}C-pyruvate, ^{13}C-lactate, and ^{13}C-lactate/^{13}C-pyruvate signal. The original resolution is indicated by a white box. **(B)** The ^{13}C slice orientation and thickness is indicated by yellow lines in the sagittal (top) and coronal (bottom) ^{1}H MRI. **(C)** Anatomical ^{1}H MRI showing regions of interests (white) used to calculate the voxel normalized ^{13}C NMR spectra (**D**) and dynamic ^{13}C metabolite signals (**E**): Tumor on gland 8 and 2, and heart (top to bottom). **(F)** Histology of the breast tumors with different staining indicating the structure (H&E, left), apoptosis (TUNEL, center), and proliferation (Ki67, right).

Lactate-to-Pyruvate Ratios and Kinetic Rate Constants

The lactate-to-pyruvate ratios were calculated by integrating the area under the time-curve and/or spectra of the respective region of interest (ROI). For two mice (B, D) only single-time-point spectral data was recorded. The spectral integration ranges were: Pyruvate [169 ppm, 174 ppm], lactate [183 ppm, 185 ppm], noise [161 ppm, 163.5 ppm] + [186 ppm, 193 ppm]. The area under the curve was determined by integration of the first 10 frames (≈40 s) for pyruvate, 15 frames (≈60 s) for lactate. The noise was determined from the last 20 frames (≈80 s). In some ROIs, the pyruvate or lactate signal was not significantly larger than the noise, leading to unrealistic values that were excluded by adding a clause (SNR > 1.05).

As shown in figure S11, in every mouse the lactate/pyruvate level of the hypointense tumor (T_2w-) is elevated in comparison to the hyperintense tumor (T_2w+) or the heart or blood vessel.

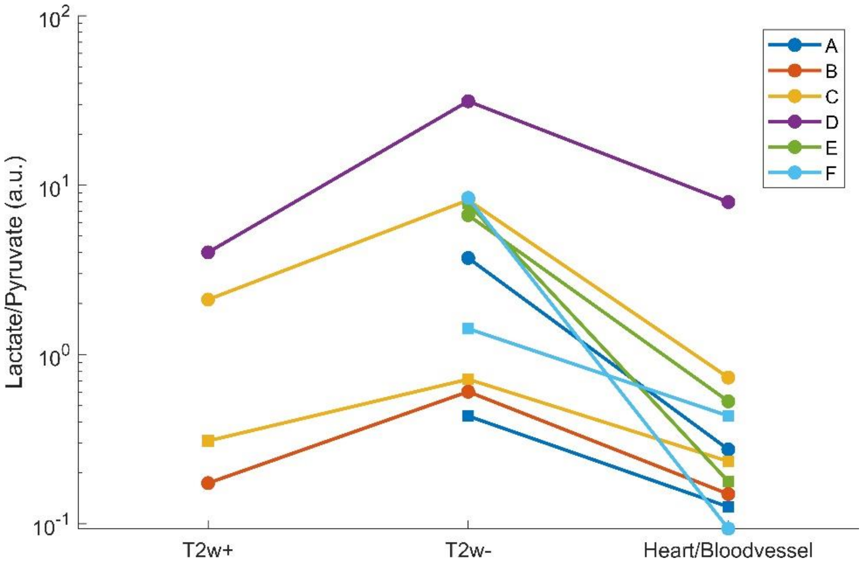

Figure S11. Lactate-to-pyruvate ratios of different structures in each mouse: Hyperintense tumor regions in the T_2w MRI (T_2w+), hypointense tumor regions (T_2w-), and heart and/or vasculature (depending on the mouse). The lactate and pyruvate values were calculated by integration of the area under the time-curve (■) or the spectrum (●) with noise subtracted.

The kinetic pyruvate-to-lactate rate constants k_{PL} were determined for the dynamically acquired data (mice A, C, E, and F) using the following differential equations to model the conversion between pyruvate (P) and lactate (L):

$$\frac{dP}{dt} = -(k_{PL} + \rho_P) \cdot P + k_{LP} \cdot L$$

$$\frac{dL}{dt} = -(k_{LP} + \rho_L) \cdot L + k_{PL} \cdot P$$

Here, P and L represent the ^{13}C signals of pyruvate and lactate, respectively. k_{PL} is the rate constant for pyruvate-to-lactate conversion, and k_{LP} the reverse rate constant for lactate-to-pyruvate conversion, which was assumed negligible [8]. ρ_P and ρ_L are the relaxation terms for pyruvate and lactate, respectively. To model the delivery and uptake of pyruvate, the pyruvate input function was represented by a smoothed Heaviside function:

$$H(t, \sigma) = \frac{A}{2} \cdot \left(1 + \frac{\pi}{2} \cdot arctan\left(\frac{t - t_0}{\sigma}\right)\right)$$

where A is the amplitude, σ is the sharpness of the step function, and t_0 is the time of the pyruvate bolus arrival. This input function leads to the total time derivative for the pyruvate signal:

$$\frac{dP_{total}}{dt} = \frac{dP}{dt} + \frac{dH}{dt}$$

The system of equations was numerically solved to fit the experimentally measured lactate and pyruvate signals, allowing the extraction of the kinetic rate constant k_{PL} for each region of interest (tumor regions and heart/blood vessels). The fitting was performed using the `LSQCURVEFIT` function in MATLAB, which minimizes the sum of squared residuals between the measured dynamic pyruvate and lactate signal curves and the modeled data.

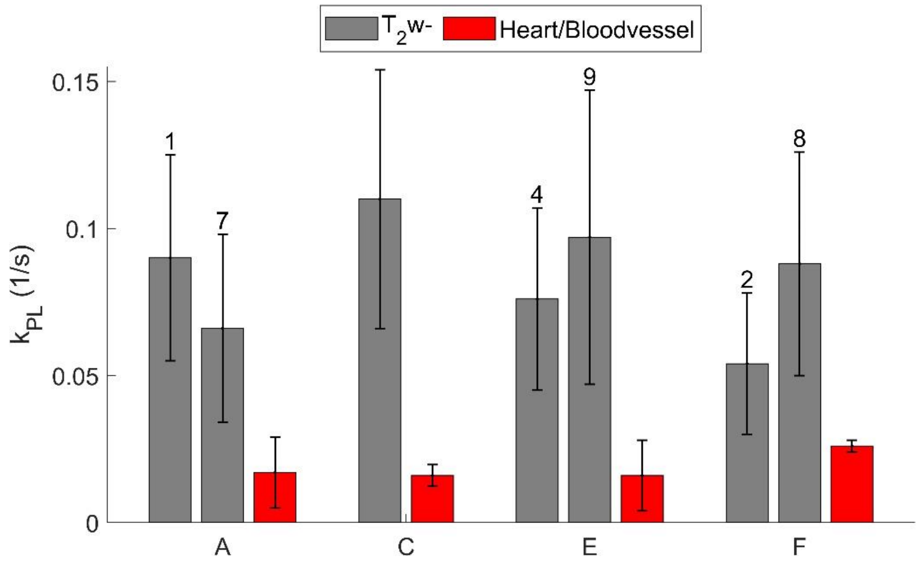

Figure S12. Kinetic pyruvate-to-lactate rate constants k_{PL} of T_2w-MRI hypointense tumor regions (T_2w-, grey) and heart/blood vessel (red) reported for the four mice measured with dynamic ^{13}C-CSI.

	ROI	Lactate/Pyruvate (a.u.)	k_{PL} (1/s)
Mouse A	Gland 1 tumor	8,19	0,090(35)
	Gland 2 tumor	1,98	-
	Gland 7 tumor	0,971	0,066(32)
	Legs*	2,59	0,019(15)
	Heart	0,257	0,017(12)
Mouse B	Gland 7 (healthy)	0,19	static
	T2w- tumor	0,603	
	T2w+ tumor	0,174	
	Blood vessel	0	
	Heart	0,3	
Mouse C	T2w+ tumor	2,11	-
	T2w- tumor	8,16	0,111(44)
	brain	2,99	0,075(29)
	Blood vessel	0,731	0,0159(37)
Mouse D	T2w+ tumor	4,01	static
	Necrosis	inf	
	T2w- tumor	31,3	
	Brain	67,5	
	Blood vessel	7,95	
Mouse E	Gland 9 tumor	6,65	0,097(50)
	Gland 4 tumor	inf	0,076(42)
	Blood vessel	0,529	0,016(12)
Mouse F	Gland 8 tumor	13,6	0,088(38)
	Gland 2 tumor	3,2	0,054(24)
	Heart	0,0937	0,0258(20)

Table S1. Lactate-to-pyruvate ratios and kinetic pyruvate-to-lactate rate constants k_{PL} of each region of interest (ROI) and mouse. The lactate and pyruvate signals were quantified by integrating the spectra of the corresponding ROI. The k_{PL} values were calculated by fitting of the dynamic ^{13}C metabolite signals. Note that in regions where the pyruvate signal approaches the noise level, the calculated ratios may have increased variability. Additionally, in some regions, the metabolite dynamics did not sufficiently stand out from the noise floor, making successful fitting impossible. * The signal assigned to the leg likely originates from the adjacent tumors.

Non-interpolated images

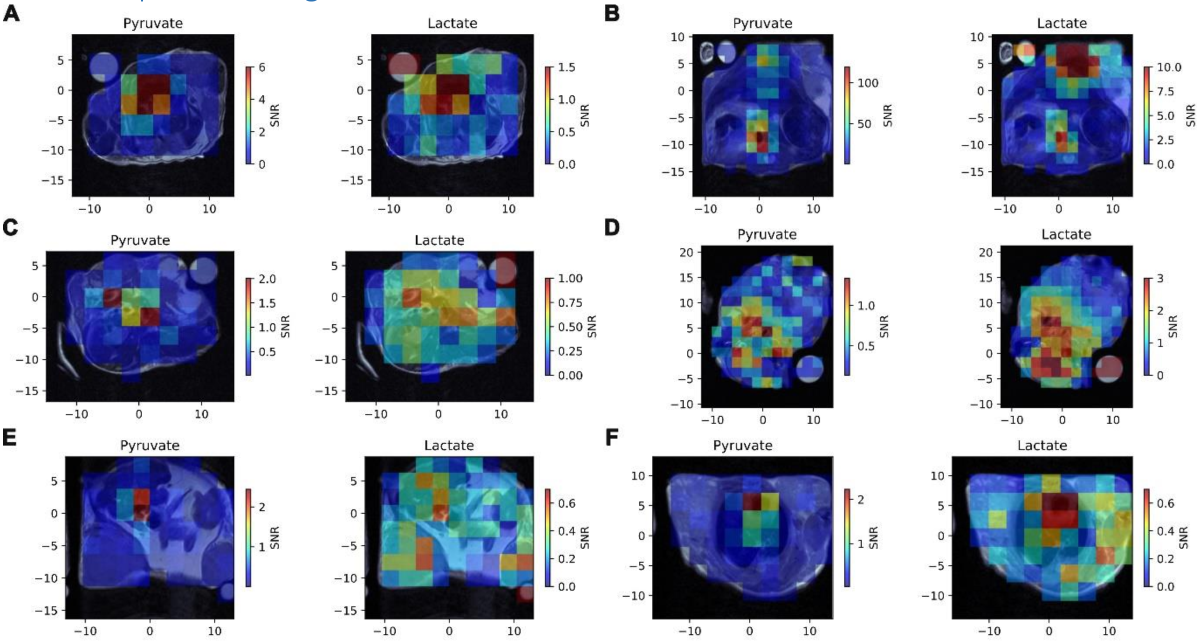

Figure S13. Non-interpolated ^{13}C pyruvate and lactate images superimposed with anatomic axial ^{1}H images of all measured mice **(A-F).**

Histology of tumors

The fixed tumors were applied to an increasing alcohol series (2x 70% EtOH, 80% EtOH, 2x 95% EtOH & 3x 100% EtOH for 1h each), Xylene (2x for 2h) and finally paraffin (2x for 2h), in which the tumors were embedded. Tissue sections of 5 µm were cut, transferred on a slide and used for different stainings.

H&E staining

For deparaffination, tumor sections were applied to Xylene (4x for 5 min.) and a decreasing alcohol series (3x 100% EtOH, 96% EtOH, 2x 70% EtOH & 2x 50% EtOH for 1 min. each) and finally distilled water. To stain the nuclei blue, tissue sections were stained with Mayers Haemalaun (Sigma-Aldrich) for 6-8 minutes and washed with water for 15 minutes. Afterwards, the cytoplasm was stained red by Eosin (Merck) for 1 minute and rinsed in 50% EtOH. The tissue sections were applied to an increasing alcohol series (2x 50% EtOH, 2x 70% EtOH, 90% EtOH, 96% EtOH for 1 min. each & 3x 100% EtOH for 2 min. each) and Xylene (3x for 5 min. &1x for 10 min.). Tissue sections were embedded in Entellan (Sigma-Aldrich) and dried overnight. Images were taken by the BZ-9000 microscope (Keyence) and merged with the BZ-II Analyzer software (Keyence).

TUNEL staining for visualization of cell death

For visualization of dead cell areas in the tumors, tumor sections were applied to Xylene (3x for 5 min.) and a decreasing alcohol series (2x 100% EtOH, 96% EtOH & 70% EtOH for 3 min. each) and finally Phosphate Buffered Saline (PBS: 1 g KCl, 1 g KH2PO4, 40 g NaCl & 7.1 g Na2HPO4 in 1 l ddH2O) with 0.2% Tween®20 (PBST) for 5 minutes. Antigen retrieval is performed by application of Proteinase K (20 µg/ml in PBST) for 15 minutes, followed by rinsing in ddH2O (2x for 2 min.). Endogenous peroxidase was blocked by 3% H2O2 & 10% MetOH in ddH2O for 20 minutes and again washed in ddH2O (2x for 5 min.).

To stain for cleaved DNA indicating dead cell areas in the tissue, the ApopTag® Peroxidase In Situ Apoptosis Detection Kit (Millipore) was used. Here, tissue sections were incubated with Equilibration Buffer for 10 seconds, followed by TdT Enzyme (1:6 in Reaction Buffer) at 37 °C in a humid chamber for 1 hour. Afterwards, the slides were incubated with Stop/Wash Buffer (1:35 in ddH2O) for 10 minutes and rinsed in PBST (3x for 1 min.). Next, Anti-Digoxigenin-Peroxidase antibody was applied to the sections for 30 minutes, followed by washing with PBST (4x for 2 min.). To visualize the dead tumor areas, 3,3´-diaminodbenzidine (DAB) substrate (Roche) was applied for 30 minutes and washed with ddH2O (3x for 1 min. & 1x for 5 min.). DAB is catalyzed by the peroxidase enzyme coupled to the Anti-Digoxigenin antibody to a brown product resulting in a brownish stain of the dead tumor areas. To stain the nuclei blue, tissue sections were stained with Mayers Haemalaun as described above and embedded in Aquatex (Merck). Images were taken by the BZ-9000 microscope (Keyence) and merged with the BZ-II Analyzer software (Keyence). Quantification of the dead tumor area was done using ImageJ software with the package Colour Deconvolution 2 (Fiji, National Institutes of Health).

As positive control, a tumor section was incubated in DN buffer (3 ml 1M Tris base pH 7.2, 400 µl 1 M MgCl2 & 1.54 mg Dithiothreitol in 100 ml ddH2O) for 5 minutes after antigen retrieval, followed by DNase I (10U/ml in DN buffer) incubation for 10 minutes to disrupt the DNA. The section was washed with ddH2O (5x for 3 min.) and afterwards applied to TUNEL staining again.

Immunohistochemistry

Tumor sections were applied to Xylene (4x for 5 min.) and a decreasing alcohol series (2x 100% EtOH for 5 min., 99% EtOH, 96% EtOH, 70% EtOH & 50% EtOH for 1 min. each) and finally ddH2O. Antigen retrieval is performed by boiling tumor section in 0.01 M citrate buffer pH 6

(9 ml 0.1 M citric acid & 41 ml 0.1 M sodium citrate in 450 ml ddH2O) for 20 minutes. After the slides cooled down for about 15 minutes, they were washed in ddH2O for 2 minutes. Endogenous peroxidase was again blocked by 3% H2O2 & 10% MetOH in ddH2O for 20 minutes, followed by rinsing in PBST for 2 minutes.

To stain for proliferating cells or endothelial cells surrounding blood vessels within the tumor tissue, a specific primary antibody was used targeting Ki67 (Proliferation; Abcam: ab15580; 1:200 in PBST) or CD31 (endothelial cells; Abcam: ab281583; 1:500 in PBST). First, tissue sections were incubated with Blocking Solution (VECTASTAIN® ABC-HRP Kit, Peroxidase (Rabbit IgG); Vector Laboratories) in a humid chamber for 30 minutes, followed by primary antibody in a humid chamber at 4°C overnight. Afterwards, the slides were washed with PBST (3x for 5 min.) and incubated with the secondary antibody provided by the kit (1:200 in Blocking Solution) for 4d according to the manual of the kit (ABC) was applied to the sections for 45 minutes and washed with PBST (3x for 5 min.). For visualization, again 3,3´-diaminodbenzidine (DAB) substrate (Roche) was applied for 8 minutes for Ki67 or 1 minute for CD31 and reaction was stopped by applying ddH2O. To stain the nuclei blue, tissue sections were stained with Mayers Haemalaun for 2-8 minutes and washed as described above and embedded in Aquatex (Merck). Images were taken by the BZ-9000 microscope (Keyence) and merged with the BZ-II Analyzer software (Keyence). Quantification of the proliferative (Ki67+) tumor area was done using ImageJ software with the package Colour Deconvolution 2 (Fiji, National Institutes of Health).5 minutes, followed by rinsing in PBST (3x for 5 min.). Next, Avidin-Biotin-Complex prepared according to the manual of the kit (ABC) was applied to the sections for 45 minutes and washed with PBST (3x for 5 min.). For visualization, again 3,3´-diaminodbenzidine (DAB) substrate (Roche) was applied for 8 minutes for Ki67 or 1 minute for CD31 and reaction was stopped by applying ddH2O. To stain the nuclei blue, tissue sections were stained with Mayers Haemalaun for 2-8 minutes and washed as described above and embedded in Aquatex (Merck). Images were taken by the BZ-9000 microscope (Keyence) and merged with the BZ-II Analyzer software (Keyence). Quantification of the proliferative (Ki67+) tumor area was done using ImageJ software with the package Colour Deconvolution 2 (Fiji, National Institutes of Health).

Vascularization/CD31 stain images

Direct measurements of tissue perfusion were not conducted in this study. However, tumor vascularization was assessed through histological staining of endothelial cells (CD31) in the tumor tissue. The staining revealed the presence of blood vessels in various tumor regions (see figure S14), with no apparent differences between tumors or between distinct areas within the tumors. This observation indicates that blood supply was likely consistent across all tumor regions and among the tumors studied.

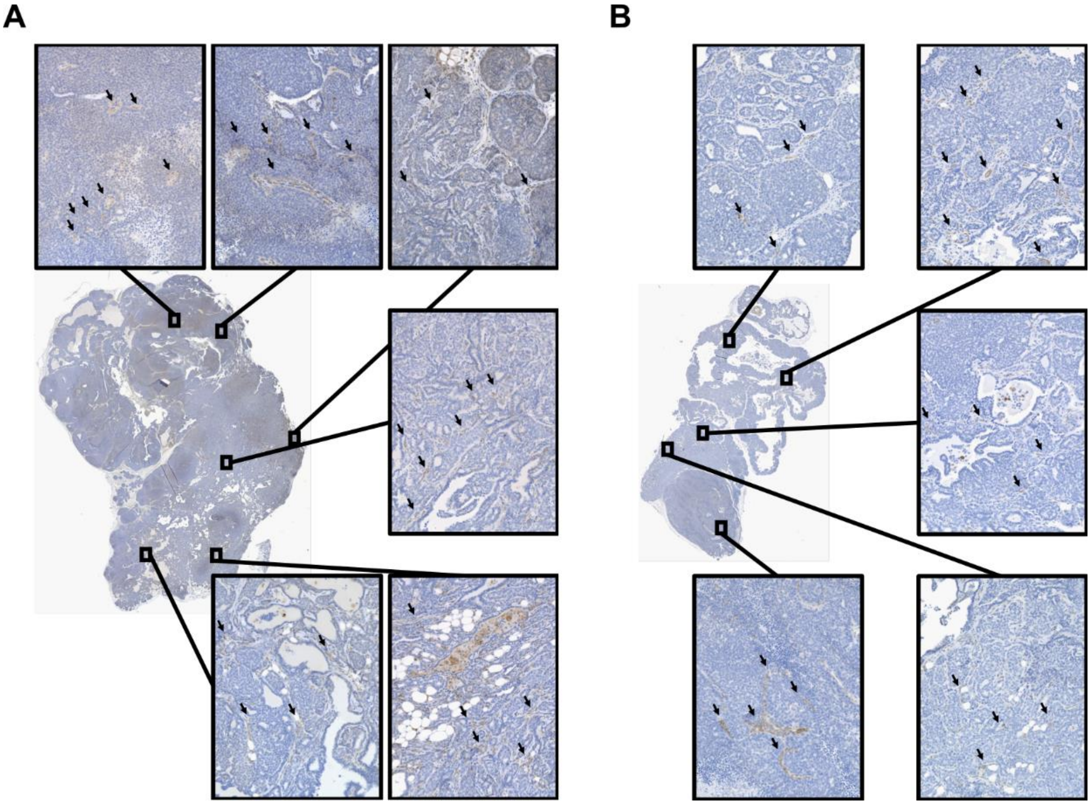

Figure S14. Histology with CD31 staining illustrating the vascularization of the breast tumors in (A) gland 2 of mouse A and (B) gland 1 of mouse B. The arrows indicate blood vessels, with no detectable differences in vascularization observed between the tumor regions.

Histological Staining Quantification

Apoptosis and proliferation were quantified by calculating the TUNEL and Ki67+ area fraction in the histological samples. While slight variations in apoptosis levels were observed between tumors, Mouse B showed a significant difference, with the T2w+ region exhibiting a higher proportion of TUNEL staining compared to the T2w- region. An opposing trend is consistently visible across tumors: regions with higher apoptosis tend to exhibit lower proliferation.

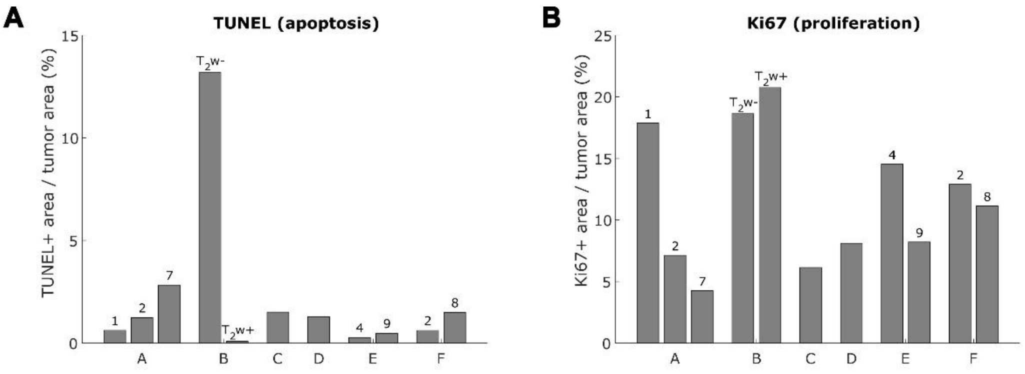

Figure S15. Quantification of (A) TUNEL staining (apoptosis) and (B) Ki67 staining (proliferation) of the histological samples as a fraction of the tumor area for all analyzed mice.

References

1. Savka R, Plenio H. Facile synthesis of [(NHC)MX(cod)] and [(NHC)MCl(CO)2] (M = Rh, Ir; X = Cl, I) complexes. Dalton Trans. 2014; 44: 891–3.

2. Hövener J-B, Bär S, Leupold J, Jenne K, Leibfritz D, Hennig J, et al. A continuous-flow, high-throughput, high-pressure parahydrogen converter for hyperpolarization in a clinical setting. NMR Biomed. 2013; 26: 124–31.

3. de Maissin H, Groß PR, Mohiuddin O, Weigt M, Nagel L, Herzog M, et al. In Vivo Metabolic Imaging of [1-13C]Pyruvate-d3 Hyperpolarized By Reversible Exchange With Parahydrogen**. Angew Chem Int Ed. 2023; 62: e202306654.

4. Kiryutin AS, Sauer G, Hadjiali S, Yurkovskaya AV, Breitzke H, Buntkowsky G. A highly versatile automatized setup for quantitative measurements of PHIP enhancements. J Magn Reson. 2017; 285: 26–36.

5. Schmidt AB, de Maissin H, Adelabu I, Nantogma S, Ettedgui J, TomHon P, et al. Catalyst-Free Aqueous Hyperpolarized [1-13C]Pyruvate Obtained by Re-Dissolution Signal Amplification by Reversible Exchange. ACS Sens. 2022; 7: 3430–9.

6. Nantogma S, Chowdhury MRH, Kabir MSH, Adelabu I, Joshi SM, Samoilenko A, et al. MATRESHCA: Microtesla Apparatus for Transfer of Resonance Enhancement of Spin Hyperpolarization via Chemical Exchange and Addition. Anal Chem. 2024; 96: 4171–9.

7. Schmidt AB, Eills J, Dagys L, Gierse M, Keim M, Lucas S, et al. Over 20% Carbon-13 Polarization of Perdeuterated Pyruvate Using Reversible Exchange with Parahydrogen and Spin-Lock Induced Crossing at 50 µT. J Phys Chem Lett. 2023; 14: 5305–9.

8. Asghar Butt S, Søgaard LV, Ardenkjaer-Larsen JH, Lauritzen MH, Engelholm LH, Paulson OB, et al. Monitoring mammary tumor progression and effect of tamoxifen treatment in MMTV-PymT using MRI and magnetic resonance spectroscopy with hyperpolarized [1-13C]pyruvate. Magn Reson Med. 2015; 73: 51–8.
